# mTOR-regulated mitochondrial metabolism limits mycobacterium-induced cytotoxicity

**DOI:** 10.1016/j.cell.2022.08.018

**Published:** 2022-09-29

**Authors:** Antonio J. Pagán, Lauren J. Lee, Joy Edwards-Hicks, Cecilia B. Moens, David M. Tobin, Elisabeth M. Busch-Nentwich, Erika L. Pearce, Lalita Ramakrishnan

**Affiliations:** 1 Molecular Immunity Unit, Cambridge Institute of Therapeutic Immunology and Infectious Diseases, Department of Medicine, University of Cambridge, Cambridge CB2 0AW, UK; 2 MRC Laboratory of Molecular Biology, Cambridge CB2 0QH, UK; 3 Department of Microbiology, University of Washington, Seattle, WA 98195, USA; 4 Max Planck Institute of Immunobiology and Epigenetics, Freiburg im Breisgau, Germany; 5 Division of Basic Sciences, Fred Hutchinson Cancer Center, Seattle, WA 98109, USA

**Keywords:** tuberculosis, mTOR, ESAT-6 mitotoxicity, mitochondrial metabolism, oxidative phosphorylation, zebrafish TB model, *Mycobacterium marinum*, *Mycobacterium tuberculosis*, macrophage death, granuloma necrosis

## Abstract

Necrosis of macrophages in the granuloma, the hallmark immunological structure of tuberculosis, is a major pathogenic event that increases host susceptibility. Through a zebrafish forward genetic screen, we identified the mTOR kinase, a master regulator of metabolism, as an early host resistance factor in tuberculosis. We found that mTOR complex 1 protects macrophages from mycobacterium-induced death by enabling infection-induced increases in mitochondrial energy metabolism fueled by glycolysis. These metabolic adaptations are required to prevent mitochondrial damage and death caused by the secreted mycobacterial virulence determinant ESAT-6. Thus, the host can effectively counter this early critical mycobacterial virulence mechanism simply by regulating energy metabolism, thereby allowing pathogen-specific immune mechanisms time to develop. Our findings may explain why *Mycobacterium tuberculosis*, albeit humanity’s most lethal pathogen, is successful in only a minority of infected individuals.

## Highlights

• mTOR deficiency confers susceptibility to mycobacterial infection
• mTOR deficiency sensitizes macrophages to mycobacterium-induced cytotoxicity
• Mycobacterial ESAT-6-induced mitochondrial damage kills mTOR-deficient macrophages
• mTOR-supported OXPHOS protects from ESAT-6-mediated virulence

## Graphical abstract

**Figure:**
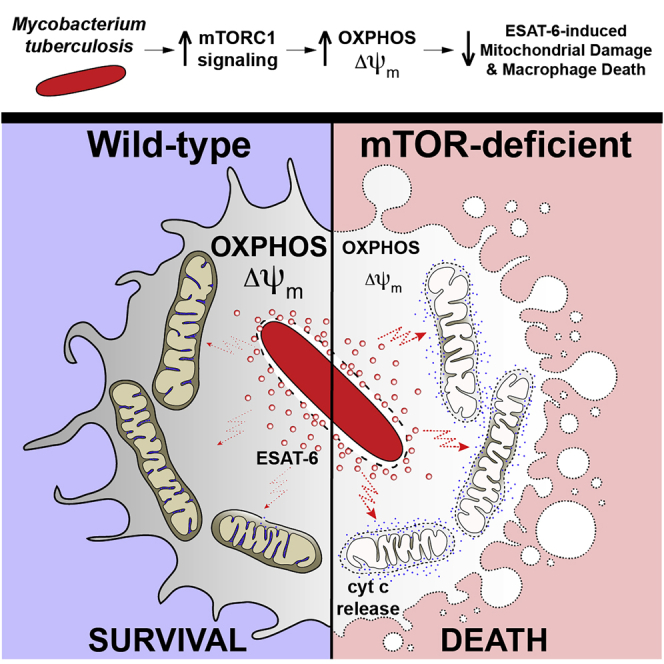

## Teaser

mTOR is an early host resistance factor in tuberculosis that prevents mitochondrial damage and protects macrophages by increasing mitochondrial energy metabolism fueled by glycolysis.

## Introduction

*Mycobacterium tuberculosis* (Mtb) induces the formation of granulomas, organized structures comprised of macrophages within which mycobacteria reside, and accessory cells (Pagán and Ramakrishnan, 2014, 2018; Ramakrishnan, 2012). The granuloma represents a key host-pathogen battleground that determines the outcome of tuberculosis (TB) infection (Pagán and Ramakrishnan, 2014; Ramakrishnan, 2012). In most individuals, the granuloma successfully clears Mtb infection, often leaving a residual, sterile, fibrotic structure as a stamp of past infection (Behr et al., 2019; Canetti et al., 1972; Feldman and Baggenstoss, 1938; Opie and Aronson, 1927; Terplan, 1951). In contrast, in the minority of people who go on to develop TB, the granuloma often becomes a mycobacterium-beneficial structure that promotes bacterial expansion and dissemination (Pagán and Ramakrishnan, 2014; Ramakrishnan, 2012). Granuloma necrosis is a pivotal pathogenic event because it delivers macrophage-resident mycobacteria into the growth-enhancing extracellular milieu (Pagán and Ramakrishnan, 2014; Ramakrishnan, 2012; Russell, 2007). Necrosis of lung granulomas with their attendant rupture into the airways also facilitates transmission, sustaining the global TB burden and promoting Mtb’s evolutionary survival (Ong et al., 2014; Ramakrishnan, 2012). Mtb’s exploitation of the granuloma begins in the innate stage of the response, enabling Mtb to gain a foothold in the host (Cambier et al., 2014a; Pagán and Ramakrishnan, 2014; Ramakrishnan, 2012). Thus, innate immune dysfunctions that facilitate this exploitation can lead to susceptibility (Cambier et al., 2014a; Pagán and Ramakrishnan, 2014; Ramakrishnan, 2012).

Zebrafish infected with *Mycobacterium marinum* (Mm), a close relative of Mtb, develop TB-like disease with necrotic granulomas (Cosma et al., 2003; Swaim et al., 2006). Mm-infected zebrafish larvae in which adaptive immunity has not yet developed also form organized granulomas that undergo necrosis (Davis et al., 2002). Thus, the zebrafish larva offers the opportunity to dissect the contribution of innate immunity in tuberculous granuloma formation and necrosis (Davis et al., 2002; Davis and Ramakrishnan, 2009; Ramakrishnan, 2013, 2020). The larva’s optical transparency and genetic tractability enables the detailed, sequential monitoring by intravital microscopy of the steps of infection, the contribution of host and pathogen determinants to them, and the consequences to outcome (Ramakrishnan, 2013). The use of unbiased genetic screens and candidate gene approaches in zebrafish larvae has shed light on granuloma biology (Ramakrishnan, 2013). Hypersusceptible zebrafish mutants displaying accelerated granuloma necrosis have identified innate immune host determinants that protect against necrosis and that are relevant to human TB (Berg et al., 2016; Clay et al., 2008; Pagán et al., 2015; Roca and Ramakrishnan, 2013; Roca et al., 2019, 2022; Tobin et al., 2010, 2012; Whitworth et al., 2021a, 2021b).

Here, we report on insights into TB pathogenesis and resistance gained from the genetic mapping and characterization of a loss-of-function mutant in mechanistic target of rapamycin (mTOR) that exhibits rapid granuloma necrosis. The mTOR pathway integrates environmental signals emanating from diverse nutrient-sensing and growth factor receptor pathways to regulate biosynthetic and metabolic processes vital for cellular development, growth, survival, and function (Liu and Sabatini, 2020). Iterative experimental approaches in the zebrafish and in human macrophages uncover the mitotoxic function of mycobacterial ESAT-6 and show that mTOR-facilitated mitochondrial metabolism serves as a highly effective innate “counter virulence” factor in TB by exerting a mitoprotective effect against this critical mycobacterial virulence factor.

## Results

### mTORC1 deficiency confers susceptibility to Mm infection in zebrafish

**Figure 1:**
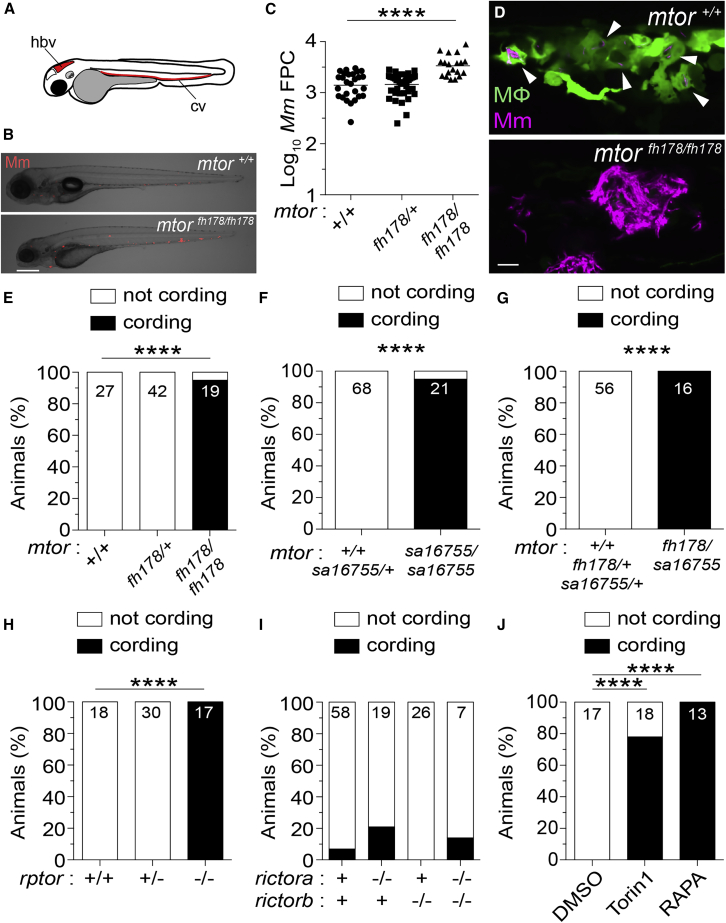
mTORC1-deficient zebrafish are hypersusceptible to Mm infection (A) Hindbrain ventricle (hbv) and caudal vein (cv) injection routes used in this study. Larvae were infected with ∼150 Mm expressing tdTomato (B), (C), and (E–J) or tdKatushka2 (D) fluorescent proteins via the caudal vein 2 days post-fertilization (dpf). (B) Overlaid micrographs of widefield mycobacterial fluorescence (Mm, red) and bright field in *mtor*^*fh178/fh178*^ or WT siblings (*mtor*^*+/+*^) 4 days post-infection (dpi). (C) Quantification of bacterial fluorescence (fluorescent pixel counts [FPCs]) in animals from *mtor*^*fh178/+*^ incross 4 dpi. Symbols represent individual animals. Horizontal lines indicate mean values. (D) Confocal micrograph optical sections of *mtor*^*fh178/fh178*^ and a WT sibling expressing *Tg(mpeg1:YFP)* 4 dpi, showing a granuloma in the WT animal and mycobacterial cording in the *mtor*^*fh178/fh178*^ animal. Mm (magenta) and macrophages (green) are shown. Arrowheads indicate intracellular Mm. (E–J) Mycobacterial cording in animals from (E) *mtor*^*fh178/+*^ incross, (F) *mtor*^*sa16755/+*^ incross, (G) *mtor*^*fh178/+*^ × *mtor*^*sa16755/+*^ cross, and (H) *rptor*^*sa11537/+*^ incross at 4 dpi, and (I) *rictora*^*sa15967/+*^*; rictorb*^*sa18403/+*^ double heterozygote incross and (J) WT animals treated with torin1 (400 nM), rapamycin (400 nM), or 0.5% DMSO (vehicle control) 5 dpi. (E–J) Numbers within columns indicate animals per group. Scale bars: 300 μm in (B) and 25 μm in (D). Statistical analyses, (C) one-way ANOVA with Tukey’s post-test and (E–J) Fisher’s exact test. Data are representative of two or more independent experiments.

The zebrafish mutant *fh178*, identified in a forward genetic screen (Tobin et al., 2010), was hypersusceptible to Mm, with larvae exhibiting increased bacterial burdens relative to wild-type (WT) and heterozygote siblings following infection into the caudal vein (cv) (Figures 1A–1C). By 4 days post-infection (dpi), *fh178* mutant granulomas had depleted their macrophages and the released mycobacteria were growing extracellularly in rope-like cords (Pagán et al., 2015; Tobin et al., 2010; Figures 1D and 1E). Mycobacterial cording, a sensitive and specific surrogate for macrophage depletion, is a readily quantifiable, binary phenotype for mapping mutants rendered hypersusceptible by granuloma necrosis (Berg et al., 2016; Tobin et al., 2010). Using polymorphic markers and the mycobacterial cording phenotype, we mapped the *fh178* mutation to a nonsense mutation in exon 24 of the *mtor* gene (see STAR Methods). We confirmed that genetic disruption of *mtor* was the cause of *fh178* hypersusceptibility by showing that animals with a nonsense mutation in exon 19 (*mtor*^*sa16755*^) (Kettleborough et al., 2013) also exhibited hypersusceptibility with cording, as did compound *fh178/sa16755* heterozygotes (Figures 1F and 1G).

mTOR is a kinase that functions in two distinct complexes, mTORC1 and mTORC2, that require the adaptors Raptor and Rictor, respectively (Liu and Sabatini, 2020). Animals with nonsense alleles of *rptor*, the gene encoding Raptor, showed similar cording to the *mtor* mutants, whereas those with nonsense alleles of *Rictor* did not (Figures 1H and 1I). Inhibition of mTOR or mTORC1 with torin1 or rapamycin, respectively (Benjamin et al., 2011), recapitulated genetic mTOR/mTORC1 deficiency with increased cording (Figure 1J). Thus, mTORC1 deficiency confers susceptibility to mycobacterial infection, linked to early granuloma breakdown. Because zebrafish larvae have not yet developed adaptive immunity, this reflects innate resistance conferred by mTOR.

### mTOR deficiency accelerates death of mycobacterium--infected macrophages

**Figure 2:**
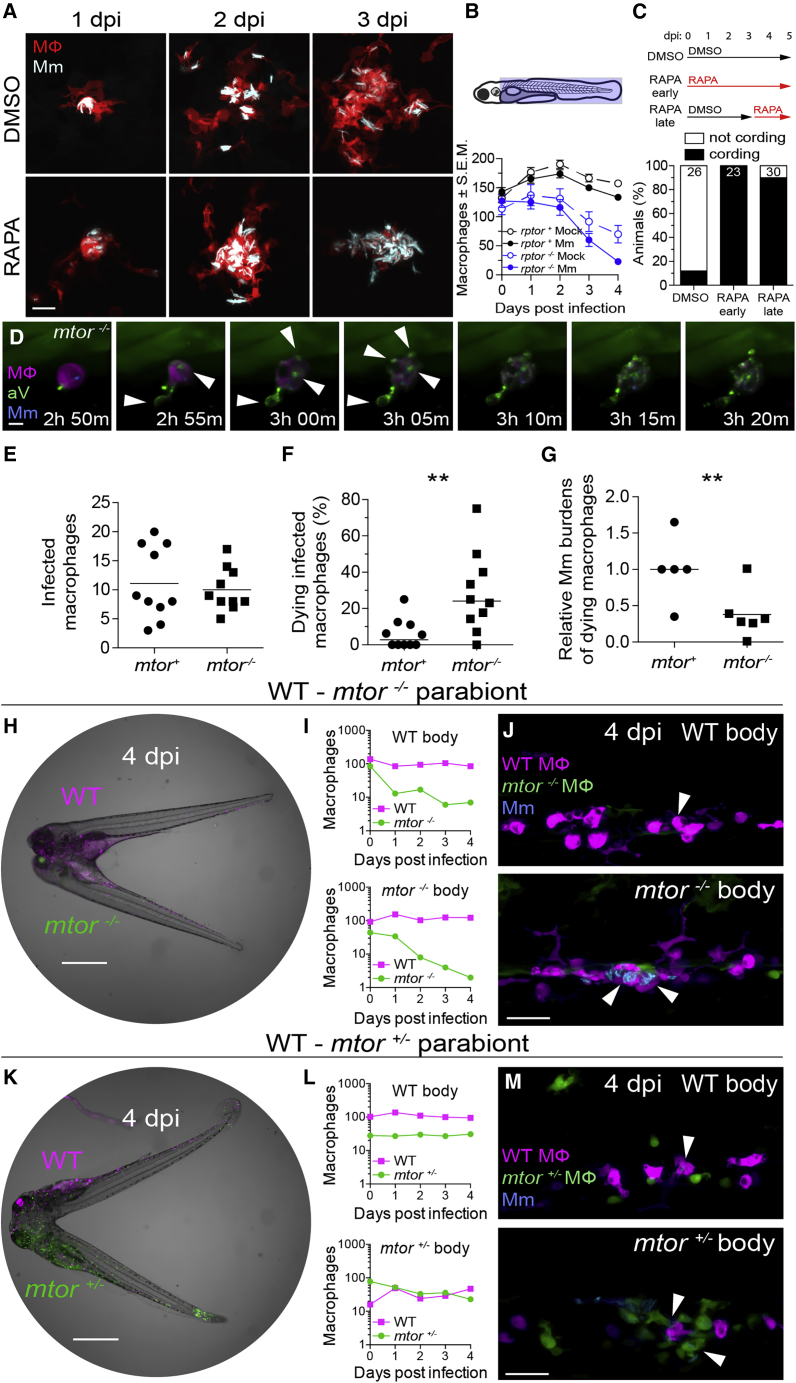
mTOR deficiency impairs macrophage development and survival and sensitizes infected macrophages to mycobacterium-induced cytotoxicity Larvae were infected with Mm expressing BFP2 (A), (E), and (I–N), mWasabi (C and D), or tdKatushka2 (F–H) fluorescent proteins via the hindbrain ventricle (A) or the caudal vein (B–N) 2 dpf. (A) Serial confocal micrographs of granulomas in *Tg(mfap4:tdTomato-CAAX)* zebrafish treated with rapamycin or DMSO. Mm (cyan), macrophages (red). (B) (Top) Macrophage counting region (shaded light blue). (Bottom) Numbers of macrophages in Mm- and mock-infected Raptor mutants and siblings expressing *Tg(mpeg1:tdTomato)*. Symbols indicate mean values for each group. Error bars show SEM. (C) (Top) Duration of rapamycin and DMSO treatments. (Bottom) Mycobacterial cording 5 dpi. (D) Time-lapse confocal micrographs of a dying infected macrophage in an *mtor*^*sa16755/sa16755*^*; Tg(mfap4:tdTomato-CAAX); Tg(ubb:secA5-YFP)* animal 2 dpi. Mm (blue), secreted annexin V-YFP (green), macrophage (magenta), annexin V^+^ blebs (arrowheads). See Video S1. (E–G) 6-h time-lapse confocal microscopy of *mtor*^*fh178/fh178*^ and mTOR-sufficient siblings expressing *Tg(mpeg1:YFP)* 2 dpi. See Video S2. (E) Absolute numbers of infected macrophages per field. (F) Percentage of dying infected macrophages per field. (G) Relative mycobacterial burdens in dying macrophages of *mtor*^*−/−*^ and mTOR-sufficient fish. Bacterial volumes were normalized to values obtained from dying cells in mTOR-sufficient controls for each imaging run. (H) Widefield micrograph of parabiotic zebrafish comprised of conjoined WT *Tg(mpeg1:tdTomato)* and *mtor*^*fh178/fh178*^*; Tg(mpeg1:YFP)* embryos 4 dpi. (I) Absolute numbers of macrophages in the WT body (top) and *mtor*^*−/−*^ body (bottom) of WT-*mtor*^*−/−*^ parabiont. (J) Maximum intensity projections of infections in the WT body (top) and *mtor*^*−/−*^ body (bottom) of a WT-*mtor*^*−/−*^ parabiont 4 dpi. (K) Widefield micrograph of WT *Tg(mpeg1:tdTomato)* and *mtor*^*fh178/+*^*; Tg(mpeg1:YFP)* parabiont 4 dpi. (L) Absolute numbers of macrophages in the WT body (top) and *mtor*^*+/−*^ body (bottom) of WT-*mtor*^*+/−*^ parabiont. (M) Maximum intensity projections of infections in the WT body (top) and *mtor*^*+/−*^ body (bottom) of a WT-*mtor*^*+/−*^ parabiont 4 dpi. Scale bars: 25 μm in (A), 10 μm in (D), 400 μm in (H) and (K), and 50 μm in (J) and (M). Horizontal lines indicate mean (E) and (G) or median (F) values. Statistical analyses, (E–G) two-tailed, unpaired Student’s t test. Time lapse data were pooled from five (E and F) or three (G) independent experiments. See also Figures S1 and S2.

At 4 dpi, WT animals had granulomas with sparse intracellular bacteria; in contrast, mTOR-deficient animals had clusters of extracellular bacteria in shapes similar to WT granulomas, suggesting that they had been in granulomas that had broken down due to macrophage death (Figure 1D, compare top and bottom). To detail the kinetics of macrophage death, we infected zebrafish larvae in the hindbrain ventricle (hbv), an acellular compartment ideal for monitoring early granuloma formation (Davis and Ramakrishnan, 2009). Animals rendered mTOR-deficient by rapamycin treatment formed granulomas similar to WT by 2 dpi, but their macrophages died by 3 dpi, leaving clumps of extracellular bacteria (Figure 2A).

We hypothesized that mTORC1 deficiency might cause granuloma breakdown through its impairment of myelopoiesis (Karmaus et al., 2017; Lee et al., 2017). The paucity of available macrophages to replenish the growing granuloma could cause its breakdown, similar to the case of myeloid growth factor colony stimulating factor-1 receptor (CSF-1R) deficiency (Pagán et al., 2015). Supporting this possibility, mTORC1 promotes myelopoiesis through CSF-1R (Karmaus et al., 2017). However, even though mTORC1-deficient animals had more macrophages at baseline than CSF-1R-deficient animals, their granulomas broke down sooner (2–4 days versus 5–7 for CSF-1R mutants) (Figure 2B; Pagán et al., 2015), suggesting that mTOR deficiency induces death of infected granuloma macrophages independently of reducing basal macrophage supply. In support of this, rapamycin treatment after formation of granulomas caused their rapid breakdown (Figure 2C). For further confirmation, we used time-lapse microscopy to capture in real-time the death of infected macrophages. If mTOR-deficient granulomas are breaking down due to reduced macrophage replenishment, then dying mTOR mutant macrophages should have bacterial burdens similar to or greater than WT. To assess bacterial burdens in dying macrophages, we used blue fluorescent Mm to infect mTOR-deficient animals and their WT siblings, which had red fluorescent macrophage membranes. All animals were also transgenic for a ubiquitously expressed secreted annexin V tagged with yellow fluorescent protein (*Tg(ubb:secA5-YFP)*), which accumulates on the surface of cells undergoing apoptosis and other modes of regulated cell death (Bendall and Green, 2014). We monitored macrophage death by the appearance of annexin V-YFP labeling of the plasma membrane and membrane blebs followed by the loss of tdTomato fluorescence reflecting plasma membrane disintegration (Figure 2D; Video S1). In 2 dpi animals, over a 4.5-h imaging period that captured similar numbers of infected macrophages in mTOR-deficient and WT siblings, 6-fold more mTOR-deficient macrophages died (Figures 2E and 2F; Video S2). Importantly, mTOR-deficient macrophages died with lower bacterial burdens than WT (Figure 2G). This finding ruled out numerical macrophage defects as the cause of macrophage death and showed that mTOR-deficient macrophages died because they were “intolerant” of mycobacterial infection.

Video S1. Annexin V labeling of a dying, Mm-infected macrophage in an mTOR mutant animal, related to Figure 2 Time-lapse confocal microscopy of a dying, infected macrophage in an *mtor*^*sa16755/sa16755*^*; Tg(mfap4:tdTomato-CAAX); Tg(ubib:secA5-YFP)* animal 2 dpi. Mm (blue), secreted annexin V-YFP (green), and macrophage (magenta).

Video S2. Death of Mm-infected macrophages in mTOR mutant animals, related to Figure 2 Time-lapse confocal microscopy of *mtor*^*fh178/fh178*^ animals and mTOR-sufficient siblings expressing *Tg(mpeg1:YFP)*, 2 dpi. Mm (magenta) and macrophages (green).. Arrows indicate dying infected macrophages.

To determine whether mTOR’s protective effect was macrophage-intrinsic, we compared mTOR mutant and WT macrophages in the same environment by creating WT-mTOR mutant parabionts with differentially labeled macrophages, red and yellow fluorescent in WT and mTOR mutants, respectively, and infecting both through their respective caudal veins (Figure 2H). By 4 dpi, mTOR mutant macrophages had been depleted by more than 90% on both WT and mTOR mutant sides of the parabionts, whereas WT macrophages persisted equally on both sides (Figure 2I). The depletion of mTOR mutant macrophages in these animals should not lead to Mm cording because the complement of WT macrophages should be able to engulf the infected mTOR mutant corpses and keep Mm intracellularly. By 4 dpi, as predicted, Mm was only found inside the WT macrophages (Figure 2J). In mTOR heterozygote-WT parabionts, mTOR heterozygote yellow fluorescent macrophages survived as well as WT red fluorescent macrophages, ruling out *Tg(mpeg1:YFP)* expression as an artifactual cause of macrophage depletion of mTOR mutant macrophages (Figures 2K and 2L). Thus, mTOR confers early cell-intrinsic protection against *Mycobacterium*-induced death.

### Infected macrophage death in mTOR deficiency is associated with reduced mitochondrial membrane potential

**Figure S1:**
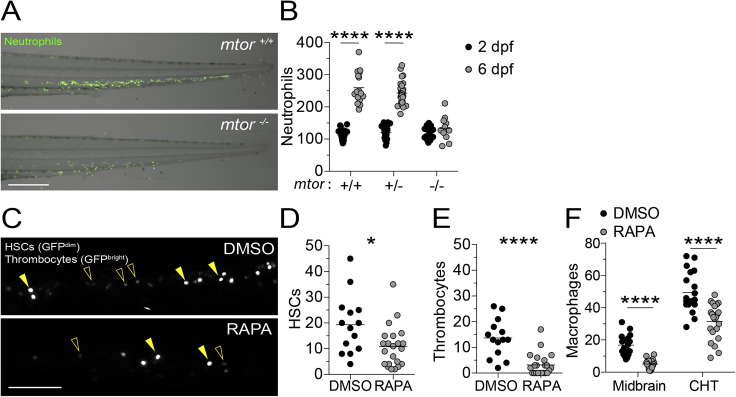
mTOR deficiency impairs hematopoiesis in zebrafish, related to Figure 2 (A) Overlaid widefield fluorescence and bright-field micrographs of an *mtor*^*fh178/fh178*^ animal and wild-type sibling expressing the neutrophil-specific fluorescent reporter *Tg(lysC:EGFP)* 6 dpf (Hall et al., 2007). (B) Numbers of neutrophils in the caudal hematopoietic tissue (CHT) of animals from *mtor*^*fh178/+*^*; Tg(lysC:EGFP)* incross 2 and 6 dpf. (C–F) Zebrafish embryos were manually dechorionated and treated with 400 nM rapamycin or 0.5% DMSO on 1 dpf to block primitive and intermediate waves of hematopoiesis (Clements and Traver, 2013). (C) Confocal micrographs of the CHT of *Tg(cd41:GFP)* zebrafish at 2 dpf. Hematopoietic stem cells (HSCs, open arrowheads) and thrombocytes, nucleated counterparts of platelets in non-mammalian vertebrates, (filled arrowheads) were identified by low versus high level expression of *cd41:GFP*, respectively (Lin et al., 2005; Ma et al., 2011). (D) Numbers of HSCs in the CHT of 2 dpf animals. (E) Numbers of thrombocytes in the CHT of 2 dpf animals. (F) Numbers of macrophages in the midbrain and CHT of *Tg(mpeg1:YFP)* zebrafish 2 dpf. Scale bars: 300 μm in (A) and 100 μm in (C). (B and D–F) Symbols represent individual animals. Horizontal lines indicate means. Statistical analyses, (B) two-way ANOVA with Tukey’s post-test and (D–F) unpaired Student’s t test.

Impairment in nutrient sensing pathways caused by mTOR-deficiency could sensitize cells to autophagic death or mitochondrial apoptosis (González et al., 2020; Green and Levine, 2014; Hosoi et al., 1999; Muthukkumar et al., 1995). To assess autophagic death, we created zebrafish deficient in the essential autophagy protein ATG12 (Bento et al., 2016). ATG12-deficient animals had defective autophagosome formation as evidenced by reduced LC3 aggregation (Figures S1A and S1B). However, ATG12 deficiency did not prevent death of mycobacterium-infected macrophages in rapamycin-treated animals (Figure S1C). To assess mitochondrial apoptosis, we used caspase-9 deficient zebrafish mutants (Galluzzi et al., 2016). Capase-9 mutants had the expected defect in developmental apoptosis as reflected by reduced dead cell debris in the brain at 3 days post-fertilization (dpf) (Figures S1D and S1E). However, rapamycin treatment induced death of their infected macrophages (Figure S1F). Thus, mTOR deficiency kills infected macrophages independent of inducing autophagy or mitochondrial apoptosis.

**Figure S2:**
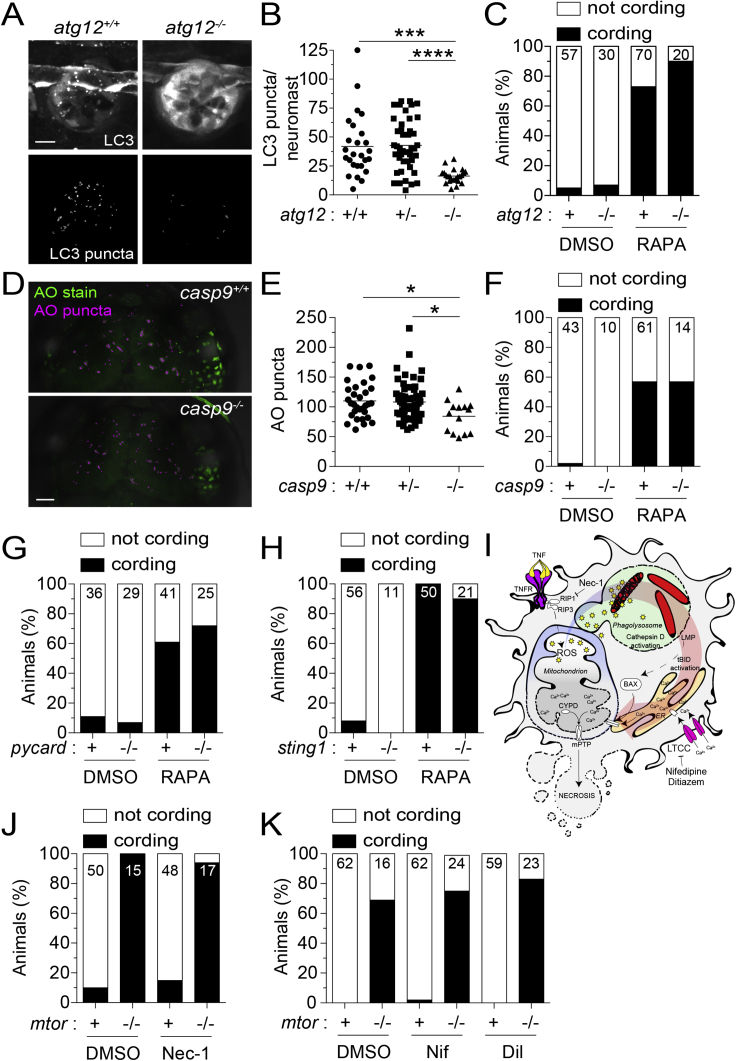
Inhibition of autophagic cell death, mitochondrial apoptosis, or TNF-associated necrosis does not prevent mycobacterium-induced macrophage death in mTOR-deficient animals, related to Figures 2 and 3 (A) Confocal micrographs of LC3 aggregation in neuromasts, clusters of mechanosensory cells of the fish lateral line, from *atg12*^*sa42684*^ incross fish expressing *Tg(CMV:lc3b-GFP)* 5 dpf. GFP fluorescence (top) and surface-rendered puncta (bottom) are shown. Scale bar, 10 μm. (B) Number of LC3 puncta per neuromast. (C) Cording in rapamycin- or DMSO-treated *atg12*^*sa42684*^ incross fish 5 dpi. (D) Confocal micrographs of acridine orange (AO) staining (green) and surface-rendered puncta (magenta) in the midbrain of *casp9*^*sa11164*^ incross fish 3 dpf. Scale bars, 50 μm. (E) Number of AO puncta in the midbrain. (F) Cording in rapamycin- or DMSO-treated *casp9*^*sa11164*^ incross fish 5 dpi. (G) Cording in rapamycin- and DMSO-treated *pycard*^*w216/w216*^ (Asc-deficient) animals and siblings 4 dpi. (H) Cording in rapamycin- and DMSO-treated *sting1*^*sa35634*^ (Sting-deficient) animals and WT siblings 5 dpi. (I) Necrosis pathway induced by mycobacterial infection plus excess TNF and pharmacological interventions tested. (J and K) Mycobacterial cording in *mtor*^*fh178/+*^ incross fish treated with (H) necrostatin-1, (I) nifedipine (5 μM), diltiazem (10 μM), or 0.5% DMSO 4 dpi. Symbols represent individual (B) neuromasts or (E) animals. (B and E) Horizontal lines indicate mean values. (C, F–H, J, and K) Numbers within columns indicate animals per group. (B and E) One-way ANOVA with Tukey’s post-test.

We ruled out two other modes of macrophage death commonly associated with mycobacterial infection—inflammasome-dependent, mediated through the adapter ASC, and type 1 interferon-dependent, mediated through the cytosolic DNA sensing adapter STING (Decout et al., 2021; Swanson et al., 2019). ASC or STING mutants had increased macrophage death when rendered mTOR-deficient (Figures S2G and S2H).

**Figure 3:**
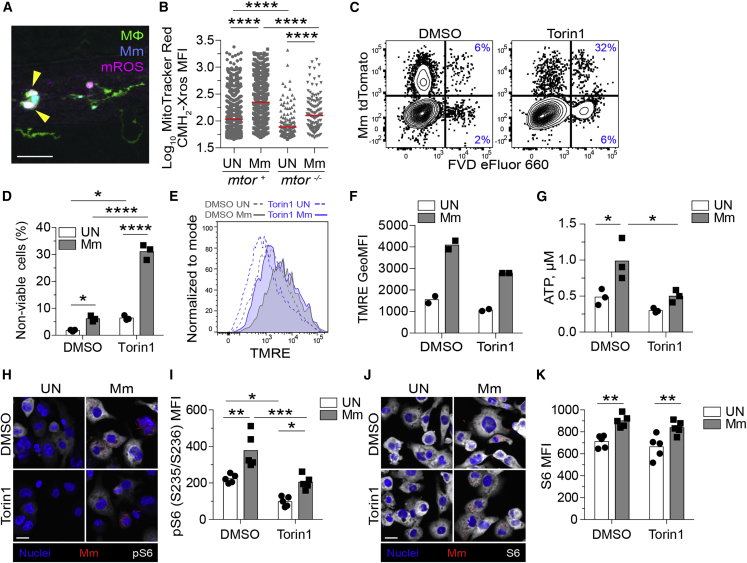
mTOR deficiency impairs basal and mycobacterium-stimulated mitochondrial metabolism in macrophages (A and B) *mtor*^*fh178/fh178*^ and mTOR-sufficient siblings expressing *Tg(mpeg1:YFP)* were infected intravenously with Mm expressing BFP2 on 2 dpf and injected intravenously with MitoTracker Red CMH_2_-Xros 1 day later. (A) Confocal micrograph illustrating mROS detection in an infected animal. Macrophages (green), Mm (blue), mROS (magenta), mROS-producing infected cells (arrowheads). Scale bar, 20 μm. (B) MitoTracker Red CMH_2_-Xros mean fluorescence intensity (MFI) in infected and uninfected macrophages of *mtor*^*−/−*^ animals and siblings at 1 dpi. Symbols represent individual macrophages. Horizontal lines indicate mean values. (C–K) THP-1 macrophages were treated with torin1 or DMSO and infected with (C, D, G, and H–K) tdTomato- or (E and F) mWasabi-expressing Mm at a multiplicity of infection (MOI) of 1 (C–F) or 3 (G and H–K). (C) Flow cytometry plots of cell viability 2 dpi. Percentages of non-viable cells (FVD eFluor 660^+^) in the infected and uninfected subpopulations are shown. (D) Quantification of non-viable cells. Symbols represent values from individual wells. Bars indicate mean values. (E) Flow cytometry histograms of TMRE fluorescence 1 dpi. (F) TMRE geometric mean fluorescence intensities (GeoMFIs) 1 dpi. Symbols represent values from individual wells. Bars indicate mean values. (G) ATP concentration per well containing 10^6^ THP-1 macrophages 1 dpi. (H–K) 1 dpi THP-1 macrophage cultures infected with tdTomato-expressing Mm (MOI = 2) were treated with torin1 or DMSO for 4 h in serum-free media.Confocal micrographs depicting Hoechst-stained nuclei (blue), Mm (red), and (H) phospho-S6^S235/S236^ or (J) total S6 staining (white). Scale bars, 20 μm. (I and K) Mean fluorescent intensity (MFI) of (I) phospho-S6^S235/S236^ and (K) total S6 staining in uninfected and infected cells. Bars indicate group means. Symbols depict average MFI per field. Statistical analyses, (B) one-way or (D), (G), (I), and (K) two-way ANOVA with Tukey’s post-test. (A), (B), and (H–K) Data are representative of two experiments. See also Figure S2.

In contrast to mTOR deficiency, where macrophages died rapidly when bacterial burdens were still low, mycobacterium-induced macrophage death typically occurs with high intracellular bacterial burdens (Amaral et al., 2019; Beckwith et al., 2020; Clay et al., 2008; Lee et al., 2011), We had previously identified a case of macrophage death associated with low mycobacterial burdens, which was mediated through a pathway activated by dysregulated tumor necrosis factor (TNF) (Roca and Ramakrishnan, 2013; Roca et al., 2019). mTOR-deficient macrophage death did not involve this pathway; inhibition of essential components, the kinase RIP1 and the L-type calcium channels, failed to rescue death (Figures S1I–S1K). Moreover, mTOR-deficient infected macrophages produced less mitochondrial reactive oxygen species (mROS) than WT ones, in contrast to macrophage death in high TNF animals, which is initiated by excess mROS (Roca and Ramakrishnan, 2013; Roca et al., 2019; Figures 3A and 3B). mTOR-deficient macrophages had lower baseline mROS and a muted infection-induced increase, which only reached baseline WT levels (Figure 3B). mTOR regulates mitochondrial metabolism (Cunningham et al., 2007; Liu and Sabatini, 2020; Morita et al., 2013; Rambold and Pearce, 2018; Schieke et al., 2006); our findings suggested that mTOR-dependent increases in mitochondrial metabolism in response to mycobacterial infection constitute a protective metabolic adaptation that prevents pathogenic macrophage death.

To explore this further, we infected the THP-1 human macrophage cell line rendered mTOR-deficient by torin1 treatment. As in the zebrafish, mTOR deficiency increased macrophage death within 1-day post-infection, as indicated by staining with the cell membrane impermeant fixable viability dye eFluor 660 (Figures 3C and 3D). Moreover, tetramethylrhodamine ethyl ester (TMRE) staining showed that infection increased mitochondrial membrane potential in an mTOR-dependent manner (Murphy, 2009; Figures 3E and 3F). Consistent with defective mitochondrial metabolism, mTOR-deficient macrophages had slightly lower baseline ATP production, again with a muted infection-induced increase that only reached baseline WT levels (Figure 3G).

We found that infection increased mTORC1 signaling, as evidenced by increased phosphorylation of the ribosomal protein S6 (Battaglioni et al., 2022; Figures 3H–3K). Torin1-treated cells had a small increase in S6 phosphorylation, consistent with residual mTOR activity (Figures 3H and 3I). Notably, this increase only reached baseline WT levels, tracking with infection-induced increases in mitochondrial metabolism (mROS and TMRE) to baseline WT levels in mTOR-deficient conditions. Together, these findings suggest that baseline mTOR-facilitated mitochondrial metabolism is insufficient to protect macrophages from mycobacterium-induced death; adaptive infection-induced mTOR activity and corresponding increases in mitochondrial metabolism are required.

**Figure 4:**
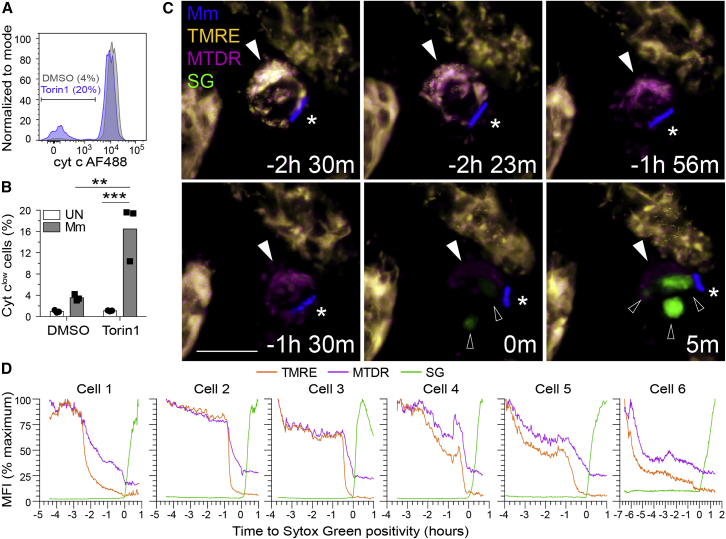
mTOR deficiency promotes mycobacterium-induced, mitochondrially mediated cell death (A and B) THP-1 macrophages were infected with (A and B) tdTomato- or (C and D) BFP-expressing Mm at MOI = 3. (A) Flow cytometry histograms of cytochrome *c* (cyt *c*) fluorescence in infected viable cells (FVD eFluor 660^−^) 7 h post-infection (hpi). Gate indicates cells that have released cyt *c*. (B) Quantification of cyt c^low^ cells 7 hpi. (C and D) Torin1-treated THP-1 macrophages were labeled with TMRE and MitoTracker Deep Red prior to imaging in the presence of Sytox Green 32 hpi. See Video S3 and Figure S2. (C) Confocal micrographs of a dying infected macrophage (filled arrowhead) surrounded by surviving uninfected macrophages. Mm (asterisk), Sytox Green (open arrowheads). Scale bars, 10 μm. (D) MFI of TMRE, MitoTracker Deep Red, and Sytox Green staining of dying infected macrophages over time. Key time-lapse frames for cell 1 are shown in (C). Statistical analyses, (B) two-way ANOVA with Tukey’s post-test.

To determine whether death of infected mTOR-deficient macrophages was due to mitochondrial damage, we assessed cytochrome *c* release by flow cytometry (Lienard et al., 2020). More mTOR-deficient infected cells had released cytochrome *c* than uninfected cells, which had released hardly any (Figures 4A and 4B). To confirm that mitochondrial damage was the cause rather than the consequence of death, we checked whether mitochondrial depolarization preceded death. Macrophages infected with blue fluorescent Mm for 32 h were stained with TMRE, which is rapidly lost from the mitochondria upon loss of membrane potential, MitoTracker Deep Red (MTDR), a mitochondrial dye that is more resistant to changes in membrane potential, and Sytox Green, a cell membrane impermeant nucleic acid dye that labels dying cells. Widespread mitochondrial membrane depolarization (near total loss of TMRE) consistently preceded plasma membrane permeabilization and cell death (Sytox positivity) (Figures 4C and 4D; Video S3). MTDR staining outlasted TMRE staining while preceding Sytox positivity. Moreover, its loss was often incomplete at the time of Sytox positivity, consistent with some retention of mitochondrial architecture at the commencement of the death process. Thus, infection induces widespread mitochondrial damage in mTOR-deficient macrophages, causing cytochrome *c* release and death. Infection-induced increases in mTOR activity are adaptive, facilitating rapid increases in mitochondrial energy production, which appear to protect against this mycobacterium-mediated lethal mitochondrial damage.

Video S3. Mitochondrial damage precedes mycobacterium-induced cell death in mTOR-deficient macrophages, related to Figure 4 8-hr time-lapse confocal microscopy of a torin1-treated THP-1 macrophage experiencing mitochondrial damage and subsequently dying (arrow). Note the sequential loss of the mitochondrial dyes TMRE (gold) and MitoTracker Deep Red (magenta) before the macrophages becomes labeled with the cell-membrane impermeant nucleic acid dye Sytox Green that was already in the well. The adjacent viable uninfected cells retained the mitochondrial dyes for the duration of the experiment.

### mTOR deficiency sensitizes macrophages to mycobacterium-induced death by impairing glycolysis-dependent OXPHOS

**Figure S3:**
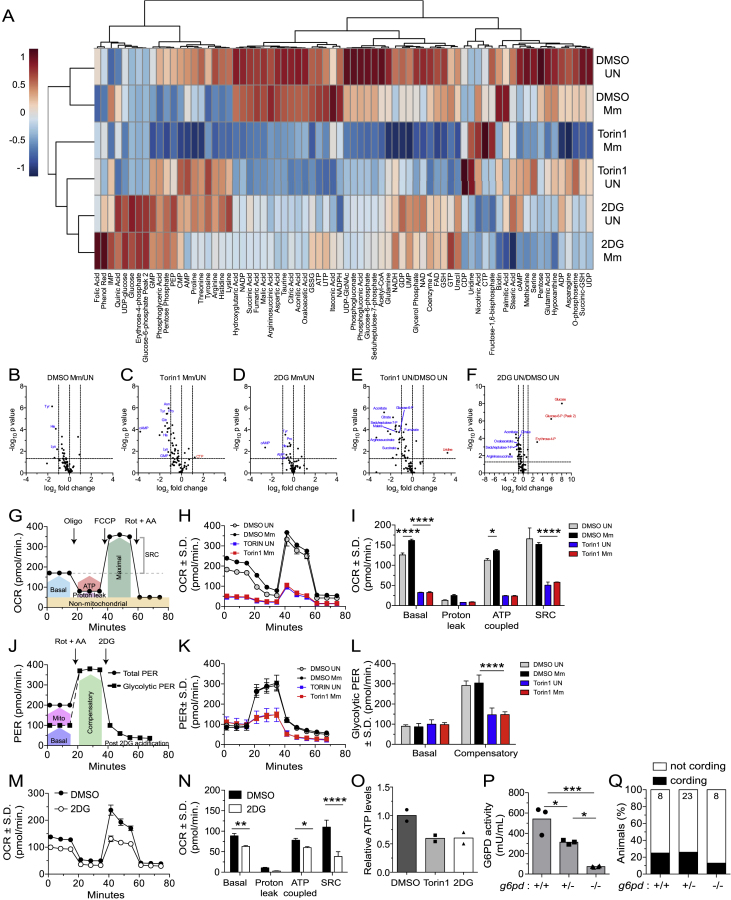
mTOR inhibition impairs glycolysis and mitochondrial metabolism, related to Figure 5 (A) Metabolite profiles of uninfected and Mm-infected THP-1 macrophages 1 dpi (MOI = 1). Cell were treated with torin1 (400 nM), 2DG (5 mM), or 0.5% DMSO for 1.5 days prior to harvest. Heat map scale indicates relative log_2_ expression levels. See also Table S1. (B–F) Volcano plots of differences in metabolite abundances induced by the indicated treatments. Dashed lines indicate fold-change and p value cutoffs. (G) Diagram of mitochondrial oxygen consumption rate (OCR) assay. (H) OCR kinetics of torin1 or DMSO-treated THP-1 macrophages 1 dpi (Mm, MOI = 4). (I) Modular analysis of mitochondrial OCR. (J) Diagram of glycolytic proton efflux rate (PER) assay. (K) PER kinetics of torin1 or DMSO-treated THP-1 macrophages 1 dpi (Mm, MOI = 4). (L) Basal and compensatory glycolytic PER. (M) OCR kinetics of uninfected THP-1 macrophages treated with 2DG or DMSO for 1.5 days. (N) Modular analysis of mitochondrial OCR. (O) Relative ATP levels in THP-1 macrophage cultures 1.5 days after treatment. (P) Glucose-6-phosphate dehydrogenase (G6PD) activity in 5 dpf animals from *g6pd*^*sa24272/+*^ incross. (Q) Cording in animals from *g6pd*^*sa24272/+*^ incross 5 dpi. Symbols represent (B–F) individual metabolites, (H, K, and M) mean values, (O) individual wells, or (P) individual animals. (I, L, and N–P) Bars indicate mean values. (H, I, and K–N) Error bars depict standard deviation. (G and J) Arrows indicate the time of compound injection. Abbreviations: rotenone plus antimycin A (Rot + AA), 2DG, oligomycin (Oligo), carbonyl cyanide 4-(trifluoromethoxy)phenylhydrazone (FCCP), compensatory glycolysis (Comp), spare respiratory capacity (SRC). Statistical significance, (B–F) unpaired Students’ t test, one-way ANOVA with (I, L, and N) Sidak or (P) Tukey’s post-tests.

Consistent with mTOR regulating a number of metabolic pathways that regulate mitochondrial metabolism, mTOR inhibition of THP-1 cells caused broad reductions in glycolysis, pentose phosphate pathways, and Krebs cycle metabolites (Düvel et al., 2010; Morita et al., 2013; Figure S3A). Correspondingly, glycolytic and respiratory capacity were reduced in mTOR-deficient cells with lower basal and ATP-coupled respiration and spare respiratory capacity (SRC) (Figures S3G–S3L). These deficits reflected compromised mitochondrial respiration and ATP production at baseline and a reduced ability to boost mitochondrial respiration in response to increased ATP demands. Moreover, mTOR deficiency blunted the small increase in mitochondrial metabolism that was apparent by 24 h post-infection (Figures S3H and S3I). At this early stage, however, infection did not alter glucose levels and glycolytic capacity, nor did it alter metabolite abundance in either WT or mTOR-deficient cells (Figures S3A–S3F and S3J–S3L). Consistent with mTOR deficiency impairing mitochondrial respiration by reducing glycolysis, selective inhibition of glycolysis with the glucose analog 2-deoxy-D-glucose (2DG) reduced Krebs cycle metabolites, mitochondrial respiration, and ATP production (Figures S3A, S3F, and S3M–S3O; Table S1).

**Figure 5:**
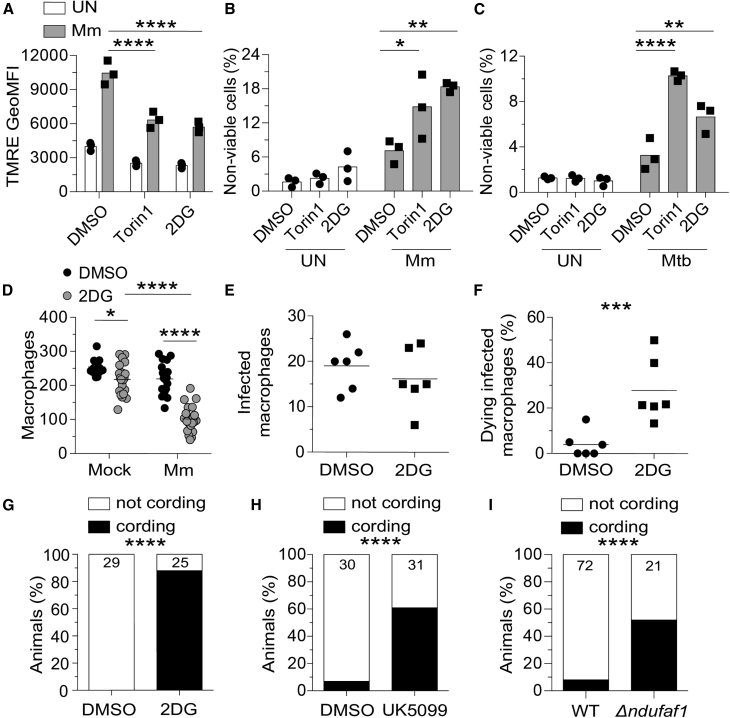
Glycolysis inhibition impairs mitochondrial metabolism and sensitizes infected macrophages to mycobacterium-induced cytotoxicity (A–C) THP-1 macrophages treated with torin1 (400 nM), 2-deoxy-D-glucose (2DG, 5 mM), or DMSO were infected with Mm expressing (A) BFP2, (B) tdTomato, or (C) Mtb expressing tdTomato (MOI = 1). (A) TMRE GeoMFI 1 dpi. (B and C) Percentage of non-viable cells (FVD eFluor 660^+^) 1 dpi. (D–I) Zebrafish were infected with ∼150 fluorescent Mm via the caudal vein. (D) 5 dpi macrophage numbers in the body of mock- or Mm*-*infected *Tg(mpeg1:YFP)* zebrafish fish treated with 50 mM 2DG or 0.5% DMSO. (E and F) 6-h time-lapse confocal microscopy of *Tg(mpeg1:YFP)* 3 dpi. (E) Absolute numbers of infected macrophages per field. (F) Percentage of dying infected macrophages per field. See Video S3. (G) Cording in wild-type (WT) animals treated with 2DG or DMSO 5 dpi. (H) Cording in WT animals treated with UK5099 (10 μM) or 0.5% DMSO 5 dpi. (I) Cording in *ndufaf1* G0 crispants and WT siblings 5 dpi. Symbols represent values from individual (A–C) and (K) wells or (D–F) animals. (A–C) Bars and (D–F) horizontal lines indicate mean values. (G–I) Numbers within columns indicate animals per group. Statistical analyses, one-way ANOVA with (A–C) Sidak, (D) Tukey post-tests, (E and F) unpaired Student’s t test, or (G–I) Fisher’s exact test. (E and F). Time-lapse data were pooled from two independent experiments. Data are representative of (A), (G), and (H), two independent experiments. See also Figure S3.

Our findings were consistent with a model where mTOR-facilitated glycolytic fueling of the Krebs cycle drives the mitochondrial energy production required to protect infected macrophages from dying. If so, then inhibition of glycolysis should phenocopy mTOR deficiency, causing selective death of infected macrophages with impaired infection-induced increase in mitochondrial membrane potential. 2DG treatment induced both phenotypes in THP-1 cells, similar to torin1 treatment (Figures 5A and 5B). mTOR and glycolysis also had a cytoprotective effect in Mtb infection. To assess this, we used mc^2^ 6206, the isogenic leucine and pantothenate auxotrophic mutant of the virulent H37Rv Mtb strain, a biosafety 2 level pathogen that elicits similar inflammatory responses and triggers diverse cell death programs (Beckwith et al., 2020; Mouton et al., 2019; Roca et al., 2019, 2022; Sampson et al., 2004, 2011). Mtb infection caused increased death of both mTOR-deficient and glycolysis-deficient THP-1 cells (Figure 5C). In the zebrafish, 2DG treatment depleted infected macrophages selectively and increased bacterial cording (Figures 5D–5G; Video S4). Thus, mTOR exerts its cytoprotective effect by supporting glycolysis both in Mm zebrafish infection and Mm- and Mtb-infected human macrophages.

Video S4. Death of Mm-infected macrophages in 2DG-treated animals, related to Figure 5 Time-lapse confocal microscopy of *Tg(mpeg1:YFP)* animals treated with 2DG or DMSO, 3 dpi. Mm (magenta) and macrophages (green). Arrows indicate dying infected macrophages.

Because glycolysis contributes to mitochondrial ATP production by supplying pyruvate to the Krebs cycle (Ryan and O'Neill, 2020), intercepting this step should also produce bacterial cording. Inhibition of the mitochondrial pyruvate carrier with UK5099 similarly increased bacterial cording (Halestrap, 1975; Figure 5H). mTOR-facilitated glycolysis also feeds the pentose phosphate pathway (Patra and Hay, 2014; Figure S2A). Zebrafish mutants deficient in glucose-6-phosphate dehydrogenase (G6PD), the rate-limiting enzyme of the pentose phosphate pathway, did not have increased bacterial cording, ruling out the contribution of this pathway to mTOR-mediated resistance (Figures S3P and S3Q). Thus, mTOR deficiency sensitizes macrophages to Mm-induced mitochondrial damage and death by impairing glycolysis-dependent oxidative phosphorylation (OXPHOS) and thereby mitochondrial energy production. Consistent with this, genetic disruption of the electron transport chain complex 1 by targeting the assembly factor NDUFAF1 increased bacterial cording (Formosa et al., 2018; Figure 5I).

### mTOR-dependent glycolysis and OXPHOS enable macrophages to resist mycobacterium ESAT-6-mediated death

**Figure 6:**
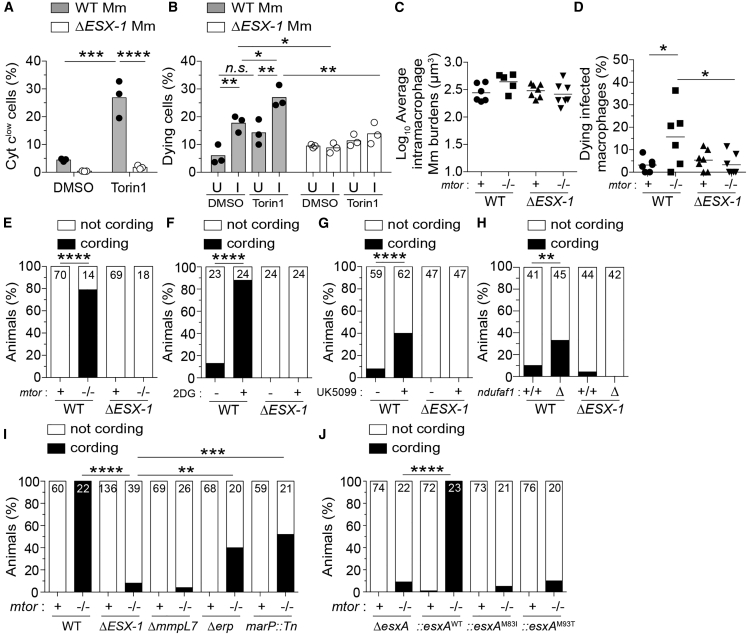
Deficiencies in mTOR, glycolysis, and OXPHOS sensitize macrophages to mycobacterial ESAT-6-dependent cytotoxicity (A) Cytochrome *c* release 7 hpi in THP-1 macrophages infected with BFP2-expressing WT or Δ*ESX-1* Mm at MOI = 3. (B) Percentage of dying cells (Sytox Green^+^) during 4-h time-lapse at 1 dpi with tdTomato-expressing WT or Δ*ESX-1* Mm at MOI = 1. Values from uninfected (U) and infected (I) cells from the same fields are shown. See Video S4. (C–J) Zebrafish were infected with dose-matched inocula of tdTomato-expressing Mm of the indicated strains via the caudal vein. (C) Intramacrophage Mm burdens at the beginning of 6-h time-lapse confocal microscopy of *mtor*^*fh178/fh178*^ and *mtor-*sufficient siblings expressing *Tg(mpeg1:YFP)* 2 dpi. See Video S5. (D) Percentage of dying infected macrophages in same experiment shown in (C). See Video S5. (E) Cording in *mtor*^*fh178/fh178*^ animals and *mtor-*sufficient siblings 4 dpi. (F and G) Cording in WT zebrafish treated with 2DG, UK5099, or DMSO 5 dpi. (H) Cording in *ndufaf1* G0 crispants and WT siblings 5 dpi. (I) Cording in *mtor*^*sa16755/sa16755*^ animals and *mtor-*sufficient siblings 4 dpi. See also Figure S3. (J) Cording in *mtor*^*fh178/fh178*^ animals and *mtor-*sufficient siblings 4 dpi with *ΔesxA* Mm complemented with WT or point mutant Mtb *esxA*. Symbols represent values from individual (A) wells, (B) imaging fields, or (C) animals. (A and B) Bars and (C and D) horizontal lines indicate mean values. (E–J) Numbers within columns indicate animals per group. Statistical analyses, (A–D) one-way ANOVA with Sidak’s post-test or (E–J) Fisher’s exact test. (B, E, and H) Data are representative of two experiments. Zebrafish time-lapse data were pooled from four experiments. See also Figure S4.

The mTOR mutant had unmasked the potential of pathogenic mycobacteria to cause lethal mitochondrial damage in infected macrophages. We hypothesized that a specific mycobacterial determinant caused this damage. Our prime candidate was ESAT-6 secretion system 1 (ESX-1)because (1) it accelerates mycobacterium-induced macrophage death in WT (mTOR-sufficient) conditions, including in the zebrafish (Conrad et al., 2017; Davis and Ramakrishnan, 2009; Gröschel et al., 2016; Simeone et al., 2012, 2021; Volkman et al., 2004), and (2) it mediates mitochondrial damage in infected macrophages (Lienard et al., 2020; Wiens and Ernst, 2016). Whereas in WT macrophages, ESX-1-dependent death requires high intramacrophage bacterial burdens (Davis and Ramakrishnan, 2009; Volkman et al., 2004), we hypothesized that mTOR-deficiency sensitizes macrophages to ESX-1-mediated mitochondrial damage, causing death at low bacterial burdens. Consistent with our hypothesis, mTOR-deficient THP-1 macrophages infected with ESX-1-deficient Mm did not have increased cytochrome *c* release nor increased death (Figures 6A and 6B; Video S5). In mTOR-deficient zebrafish infected with ESX-1-deficient Mm, infected macrophages did not die (Figures 6C–6E; Video S6). ESX-1-deficient infection also did not cause macrophage death in animals deficient in glycolysis or OXPHOS (Figures 6F–6H). Thus, a mycobacterium-induced, mTOR-dependent increase in mitochondrial metabolism specifically counters ESX-1-dependent mitochondrial damage and cell death.

Video S5. Torin1-treated THP-1 macrophages tolerate *ΔESX-1* infection, related to Figure 6 Time-lapse confocal microscopy of THP-1 macrophage treated with torin1 or DMSO and infected with WT or Δ*ESX-1* Mm, 1 dpi. Mm (magenta) and Sytox Green-labeled dying cells (green). Arrows indicate dying infected macrophages.

Video S6. Macrophages of mTOR mutant animals tolerate *ΔESX-1* infection, related to Figure 6 Time-lapse confocal microscopy of *mtor*^*fh178/fh178*^ animals and mTOR-sufficient siblings expressing *Tg(mpeg1:YFP)* infected with WT or *ΔESX-1* Mm, 2 dpi. Mm (magenta) and macrophages (green). Arrows indicate dying infected macrophages.

**Figure S4:**
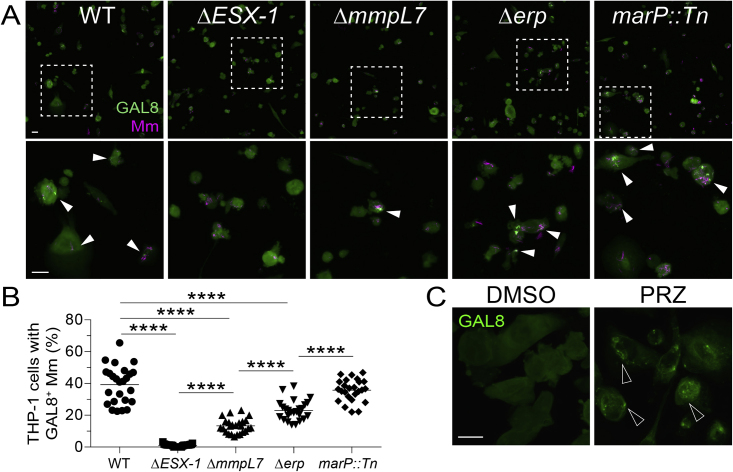
Damage of phagosomal/lysosomal compartments by ESX-1-competent mycobacteria and the drug prazosin, related to Figures 6 and 7 (A) Confocal micrographs of galectin-8 (GAL8) immunofluorescence (green) and Mm fluorescence (magenta) in THP-1 macrophages infected with the indicated Mm strains (MOI = 1) 1 dpi. The *ΔmmpL7* Mm strain is defective in PDIM transport to the myco-membrane. Bottom panels show area enclosed in dashed squares on top panels. Arrowheads indicate foci of GAL8-associated Mm. Scale bars, 25 μm. (B) Percentage of macrophages with foci of GAL8-associated Mm. Symbols represent values from individual imaging fields. Horizontal bars indicate mean values. One-way ANOVA with Tukey’s post-test. (C) Confocal micrographs of GAL8 immunofluorescence 7 h after treatment with prazosin (PRZ, 20 μM) or 0.5% DMSO. Arrowheads indicate GAL8 puncta. Scale bars, 20 μm.

ESX-1-mediated damage of *Mycobacterium*-containing phagosomes is integral to its role in virulence (Lienard et al., 2020; Simeone et al., 2012, 2021). This process is facilitated by the mycobacterial cell surface lipid phthiocerol dimycoceroserate (PDIM); Mm mutants in the PDIM transporter MmpL7 are also impaired in phagosomal damage, as indicated by reduced galectin-8 (GAL8) recruitment (Augenstreich et al., 2017; Lerner et al., 2018; Osman et al., 2020; Quigley et al., 2017; Simeone et al., 2021; Cambier et al., 2014b; Figures S4A and S4B). To determine whether ESX-1-mediated phagosomal damage was integral to its mitotoxicity in mTOR-deficient macrophages, we tested the Mm mmpL7 mutant. mmpL7 mutant Mm infection did not kill mTOR-deficient macrophages, as evidenced by the lack of cording in the animals (Figure 6I). In contrast, Mm mutants in the virulence determinants Erp and MarP, which do not mediate phagosomal damage and promote intramacrophage growth through distinct mechanisms from ESX-1, did accelerate macrophage death in mTOR-deficient animals (Berthet et al., 1998; Cosma et al., 2006; Levitte et al., 2016; Vandal et al., 2008; Figures 6I, S4A, and S4B). Thus, ESX-1-dependent phagosomal permeabilization is a pre-requisite for its cytotoxicity to mTOR-deficient macrophages.

ESX-1’s membranolytic activity has been ascribed to its major secreted substrate, ESAT-6 (6 kDa early secretory antigenic target); however, pinning down its role versus those of other ESX-1 substrates has been complicated by their co-dependency for secretion, as deletion of ESAT-6 causes loss of other ESX-1 substrates (Bao et al., 2021; Champion et al., 2014; Fortune et al., 2005). Recent work has pinpointed a specific role for ESAT-6 in phagosomal damage and virulence by identifying point mutations in ESAT-6 that allow substantial levels of secretion of ESAT-6 and other ESX-1 substrates yet cause loss of phagosomal membrane damage and/or virulence (Brodin et al., 2005; Osman et al., 2022; Zhang et al., 2016). To test the specific role of ESAT-6-induced phagosomal damage in mediating cell death in mTOR deficiency, we used *esxA* (ESAT-6) mutant Mm expressing either of two such ESAT-6 C-terminal point mutations, M83I and M93T (Brodin et al., 2005; Osman et al., 2022). Mm-ESAT-6^M83I^ and Mm-ESAT-6^M93T^ infections did not kill macrophages in mTOR-deficient zebrafish (Figure 6J). Thus, ESAT-6 causes the phagosomal damage required for the cell death induced by mTOR deficiency.

### mTOR enables infected macrophages to specifically resist ESAT-6-mediated mitochondrial damage

**Figure 7:**
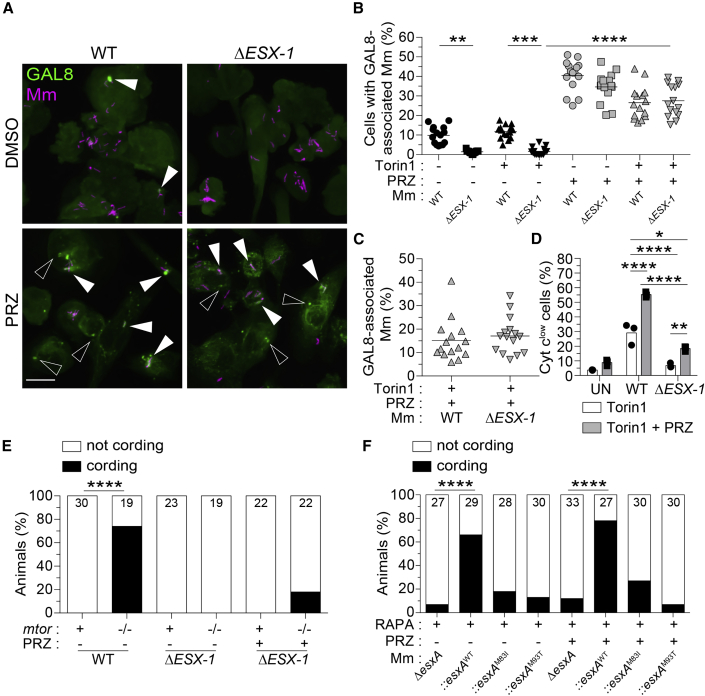
ESAT-6 mediates mitochondrial damage in mTOR-deficient macrophages downstream of its involvement in phagosomal permeabilization (A–E) Torin1- and DMSO-treated THP-1 macrophages were infected with tdTomato-expressing WT or Δ*ESX-1* Mm at MOI = 3 and treated with prazosin (PRZ, 20 μM) for 7 h. See also Figure S4. (A) Confocal micrographs of galectin-8 (GAL8) immunofluorescence (green) and Mm fluorescence (magenta) in THP-1 macrophages 7 hpi. GAL8 foci associated with Mm (filled arrowheads) or not associated with Mm (open arrowheads) are shown. Scale bar, 20 μm. (B) Percentage of macrophages with GAL8-associated Mm foci. (C) Percentage of Mm volume associated with GAL8 foci 7 hpi. (D) Percentage of cells that have released cytochrome *c* 7 hpi. (E) *mtor*^*sa16755/sa16755*^ fish and *mtor-*sufficient siblings were infected with ∼90 fluorescent Mm via the hindbrain ventricle on 2 dpf. On 1 and 2 dpi, animals were injected with ∼3 nL of 300 μM PRZ or 1% DMSO into the hbv. Graph indicates the percentage of animals with cording 3 dpi. (F) Wild-type fish treated with 400 nM rapamycin were infected with ∼180 tdTomato-expressing *ΔesxA* Mm complemented with WT or point mutant Mtb *esxA* via the hbv on 2 dpf. Animals were injected with PRZ or DMSO as indicated on (E). Graph indicates the percentage of animals with cording 3 dpi. Symbols represent values from individual (B and C) imaging fields or (D) individual wells. (B and C) Horizontal lines and (D) bars indicate mean values. (E and F) Numbers within columns indicate animals per group. Statistical analyses, (B and D) one-way ANOVA with Sidak’s post-test or (E and F) Fisher’s exact test. See also Figure S4.

mTOR-deficiency might simply sensitize phagosomes to ESAT-6-mediated damage. However, mTOR-deficient and WT macrophages had similar ESAT-6-dependent increases in phagosomal damage, showing that this was not the case and instead suggesting that mTOR-deficient macrophages were sensitized to ESAT-6-mediated mitochondrial damage (Figure 7A, upper panels and 7B, black symbols).

ESAT-6 might mediate mitochondrial damage in mTOR-deficient macrophages indirectly or directly. In the indirect case, ESAT-6 would only be required to permeabilize phagosomes, enabling host lysosomal factors or other mycobacterial determinant(s) to access and damage mitochondria. In the direct case, ESAT-6 would also be required after the phagosome has been permeabilized. To distinguish between the two, we treated THP-1 cells with prazosin (PRZ), a drug that permeabilizes endo-lysosomal compartments in myeloid cells (Kozik et al., 2020). By 7 h of treatment, PRZ had caused extensive endo-lysosomal damage in uninfected control and mTOR-deficient macrophages (Figure S4C). In infected macrophages, PRZ caused similar increases in damaged WT and ESX-1-deficient mycobacterial phagosomes (Figures 7A and 7B). Moreover, the proportion of WT and ESX-1-mutant mycobacteria associated with damaged phagosomes was similar, showing that the PRZ-induced phagosomal permeabilization could equalize access of WT and ESX-1-deficient mycobacteria to the mitochondria (Figure 7C). Consistent with increasing bacterial exposure to mitochondria, PRZ increased cytochrome *c* release from WT-infected macrophages (Figure 7D). PRZ-treated ESX-1 mutant-infected macrophages had a much smaller increase in cytochrome *c* release and significantly lower than in untreated WT macrophages (Figure 7D). The small increase in cytochrome *c* release in ESX-1-mutant infection suggests that additional factors may also play a role, for instance, PDIM. To corroborate these conclusions *in vivo*, we assessed bacterial cording in zebrafish with and without PRZ treatment. As before, mTOR deficiency caused cording of WT but not ESX-1-deficient bacteria (Figure 7E). PRZ treatment of mTOR mutants increased ESX-1-mutant cording only slightly and significantly less than WT bacteria without PRZ (Figure 7E).

Next, we tested whether ESAT-6 was responsible for the mitochondrial damage by assessing bacterial cording of ESAT-6-mutant bacteria in zebrafish with and without PRZ treatment. As with ESX-1 mutant infections, PRZ treatment did not restore bacterial cording of ESAT-6 mutants (Figure 7F). These findings implicate ESAT-6 in mediating mitochondrial damage after first damaging the phagosome to enable access. ESAT-6 possesses both phagosomal and mitochondrial damaging activity and mTOR protects specifically against the latter. Our findings highlight the role of mTOR as a “counter-virulence” factor against ESAT-6 and indicate that even a small amount of ESAT-6 is sufficient to induce cytotoxicity in mTOR-deficient macrophages. mTOR-deficient macrophages tolerate infection with mycobacteria so long as they lack functional ESAT-6.

## Discussion

Consistent with mTOR’s critical role in the development, homeostasis, and function of myeloid cells and T cells (Lachmandas et al., 2016; Powell et al., 2012; Sinclair et al., 2017; Weichhart et al., 2015), therapeutic mTOR blockade in organ transplantation and cancer has been associated with increased risk of infections, including TB (Fijałkowska-Morawska et al., 2011; Garcia and Wu, 2016; Jeon et al., 2017; Ruiz-Camps and Aguilar-Company, 2021; Tsai et al., 2007). This susceptibility has generally been ascribed to compromised adaptive immunity (Weichhart et al., 2015). We show here that mTOR deficiency results in profound, innate susceptibility to mycobacteria resulting from the rapid death of infected macrophages. This death results from catastrophic mitochondrial damage caused by mycobacterial ESAT-6. Thus, mTOR-facilitated mitochondrial metabolism represents a formidable armor against a potent mycobacterial mitotoxin. This study adds to the appreciation that while intracellular microbes can exploit host cell metabolism for growth and pathogenesis, the cells’ metabolic capabilities can also avert microbial attack (Pernas, 2021).

Our findings provide insight into the adaptive metabolic changes that occur upon mycobacterial infection. Stimulation of cultured macrophages with lipopolysaccharide (LPS), a major Gram-negative bacterial virulence determinant, induces a metabolic switch from mitochondrial OXPHOS to glycolysis under normoxic conditions (O'Neill and Pearce, 2016; Weichhart et al., 2015). This switch, which occurs through mTORC1 activation, enables macrophages to elaborate antimicrobial responses that depend on cataplerosis of Krebs cycle intermediates (O'Neill and Pearce, 2016). Whether Mtb infection induces this glycolytic switch is unclear; studies variably find that it boosts or represses glycolysis (Braverman et al., 2016; Cumming et al., 2018; Gleeson et al., 2016; Huang et al., 2018; Lachmandas et al., 2016; Olson et al., 2021; Shi et al., 2015). We find here that early in infection, Mm infection of cultured human macrophages induces OXPHOS without altering glycolysis. This is consistent with our findings in the zebrafish, where Mm- and Mtb-infected macrophages exhibit small increases in mitochondrial respiration early on (Roca et al., 2022). Although adaptive glycolytic shifts may occur later in infection, mTORC1’s role in early resistance to mycobacteria stems not from a glycolytic shift but from the boost in OXPHOS from its stimulation of glycolysis (Weichhart et al., 2015). This is demonstrated by our finding that directly inhibiting OXPHOS confers susceptibility.

How does ESAT-6 damage mitochondria? Mm and Mtb phagosomes frequently fuse to lysosomes, and contacts between lysosomes and mitochondria are proposed to facilitate metabolic exchanges between these organelles (Armstrong and Hart, 1971; Barker et al., 1997; Clemens and Horwitz, 1995; Harris et al., 2008; Levitte et al., 2016; Wong et al., 2018). The idea that mycobacterial phagolysosomes influence mitochondrial function is reinforced by the finding that mitochondria aggregate around these structures and undergo morphological changes (Jamwal et al., 2013; Mohareer et al., 2020). Given that direct contact is required for ESAT-6 permeabilization of host membranes (Conrad et al., 2017), ESAT-6 might specifically damage membranes of mitochondria that are in direct contact with permeabilized phagosomes. Indeed, ESX-1 is reported to cause some mitochondrial damage in mTOR-sufficient cells, as evidenced by reductions in mitochondrial mass, membrane potential loss, and release of cytochrome *c* and mitochondrial DNA (Chen et al., 2008; Fine-Coulson et al., 2015; Lienard et al., 2020; Pajuelo et al., 2018; Wiens and Ernst, 2016). The finding that in mTOR deficiency, infection with very few ESX-1/ESAT-6-expressing mycobacteria, likely confined to a single phagosome/phagolysosome, causes global loss of mitochondrial membrane potential suggests that there is rapid propagation of the initial localized damage. The lack of rapid repair or replacement of damaged mitochondria in the absence of mTOR-regulated biosynthetic processes may account for this (Cunningham et al., 2007; Liu and Sabatini, 2020; Morita et al., 2013; Morita et al., 2017; Rambold and Pearce, 2018; Schieke et al., 2006). Alternatively, reduced mitochondrial membrane potential has been shown to cause structural changes in the mitochondria—matrix condensation and unfolding of cristae—that facilitate cytochrome *c* release upon exposure to outer membrane disrupters like BAX family proteins (Gottlieb et al., 2003). Similar changes from mTOR deficiency could cause global mitochondrial catastrophe when subjected to ESAT-6’s mitotoxic effects. As in that report, we too find that disruption of respiration at complex I phenocopies the ESAT-6-mediated death produced by mTOR deficiency.

ESX-1 increases macrophage death, even in WT (mTOR competent) macrophages (Gröschel et al., 2016; Ramakrishnan, 2012; Augenstreich et al., 2017; Beckwith et al., 2020; Behar et al., 2010; Davis and Ramakrishnan, 2009; Srinivasan et al., 2014), with ESAT-6-mediated phagosomal damage being a pre-requisite step (Bao et al., 2021; Osman et al., 2022; Simeone et al., 2021; Srinivasan et al., 2014; Zhang et al., 2016). We show here that ESAT-6 induces phagosomal damage irrespective of whether the macrophage is mTOR-sufficient or -deficient. mTOR’s specific role is in resistance against ESAT-6-mediated catastrophic mitochondrial damage that rapidly kills the cell. We do not know whether ESAT-6 is sufficient for mitochondrial damage in mTOR deficiency. ESX-1 is responsible for plasma membrane damage in mTOR-sufficient cells and there, too, it is not clear exactly how damage is mediated—studies have variably implicated ESAT-6, other ESX-1 substrates, non-ESX-1 mycobacterial products, and excess lipoxins (Beckwith et al., 2020; Divangahi et al., 2009; Pagán and Ramakrishnan, 2018; Pajuelo et al., 2018). For the mitochondrial damage in mTOR deficiency, PDIM may facilitate ESAT-6’s role in addition to facilitating ESAT-6-mediated phagosomal damage.

TB is notorious for killing far more people over the millennia than any other infectious agent (Paulson, 2013). Yet, our recent epidemiological analyses show that most individuals clear Mtb infection through a combination of innate and adaptive immunity (Behr et al., 2018, 2019). Our work finds that tapping into mTOR-regulated homeostatic metabolic pathways constitutes a major host defense strategy. Curiously, these pathways protect against a specific mycobacterial virulence determinant, identifying mTOR as a counter-virulence factor against ESAT-6, where mTOR averts its catastrophic mitotoxicity to buy the host time to call in other “classical” immune defenses that can clear infection much of the time. In the minority of individuals, ESAT-6 and other mycobacterial virulence factors win out, allowing the evolutionary survival of the pathogen as well (Cambier et al., 2014a). Indeed, the modes of cell death that ESAT-6 produces in WT hosts through phagosomal or plasma membrane damage likely represent workarounds in mycobacterium’s pathogenic strategy (Beckwith et al., 2020; Chen et al., 2008; Divangahi et al., 2009; Pagán and Ramakrishnan, 2018; Pajuelo et al., 2018; Simeone et al., 2021; Zhang et al., 2021).

Therapies targeting mTOR are being explored for a number of conditions, including aging. Genetic and pharmacological mTOR inhibition can increase lifespan in yeast, worms, flies, and mice (Papadopoli et al., 2019; Saxton and Sabatini, 2017). Pilot studies of a short course of pharmacological mTOR inhibition in older human volunteers report increased responses to influenza vaccines (suggesting decreased immune senescence) and a reduction in self-reported viral respiratory infections (Mannick et al., 2014, 2018). Similarly, in lung TB patients receiving adjunctive mTOR inhibition therapy together with appropriate antimicrobial treatment had possible, transient improvement in lung function (Wallis et al., 2021). In a mouse model of severe TB, mTOR inhibition therapy induced host-beneficial or -detrimental effects depending on the treatment regimen; mTOR inhibition therapy reduced lung immunopathology in established infections when given in conjunction with an antimicrobial drug, but exacerbated lung damage and morbidity when administered alone in the early infection (Bhatt et al., 2021). Our finding that mTOR inhibitors dramatically increase susceptibility to pathogenic mycobacteria warrants caution in their use as anti-aging or immune boosting therapies in the many areas of the world with a high burden of TB.

### Limitations of the study

How ESAT-6 causes the mitochondrial damage that ultimately triggers cell death in mTOR deficiency remains to be determined. ESAT-6 may directly permeabilize the outer membrane of mitochondria in close proximity to permeabilized phagosomes or induce mitochondrial damage by cooperating with host or mycobacterial molecules with intrinsic mitotoxic activity. Furthermore, how reductions in mitochondrial membrane potential specifically increase sensitivity to ESAT-6-mediated mitotoxicity remains unclear. Identifying the molecular players in the cell death cascade and the alterations in mitochondrial structure and function in mTOR deficiency, and specifically in response to ESAT-6-mediated damage, should help to clarify these processes.

## STAR★Methods

### Key resources table

**Table:** 

REAGENT or RESOURCE	SOURCE	IDENTIFIER
**Antibodies**		

AlexaFluor 488 anti-cytochrome c (clone 6H2.B4)	BioLegend	Cat# 612308; RRID:AB_2565240
Phospho-S6 Ribosomal Protein (S235/S236) XP rabbit (clone D57.2.2E) (Alexa Fluor 647 Conjugate)	Cell Signaling Technology	Cat# 4851; RRID:AB_10695457
S6 Ribosomal Protein (clone 54D2) Mouse mAb (Alexa Fluor 647 Conjugate)	Cell Signaling Technology	Cat# 5548; RRID:AB_10707322
Goat IgG anti-human Galectin-8	R&D Systems	Cat# AF1305; RRID:AB_2137229
Donkey anti-goat IgG (H+L), Alexa Fluor 488	Thermo Fisher Scientific	Cat# A-11055; RRID:AB_2534102

**Optical-bottom tissue culture plates**		

96-well (half area) black plate with transparent bottom	Greiner Bio-One	Cat# 675090
VisiPlate 24-well black plate with clear bottom	Perkin Elmer	Cat# 1450–606
6-well No. 1.5 coverslip, 20 mm glass diameter, uncoated plate	MatTek	Cat# P06G-1.5-20-F
24-well No. 1.5 coverslip, 13 mm glass diameter, uncoated plate	MatTek	Cat# P24G-1.5-13-F

**Bacterial and virus strains**		

*M. marinum* M strain transformed with *pmsp12::BFP2*	Takaki et al., 2013	Derivative of ATCC # BAA-535
*M. marinum* M strain transformed with *pmsp12::mWasabi*	Takaki et al., 2013	Derivative of ATCC # BAA-535
*M. marinum* M strain transformed with *pmsp12::tdTomato*	Takaki et al., 2013	Derivative of ATCC # BAA-535
*M. marinum* M strain transformed with *pmsp12::tdKatushka2*	Takaki et al., 2013	Derivative of ATCC # BAA-535
*ΔESX1 M. marinum* M strain transformed with *pmsp12::tdTomato*	Pagán et al., 2015	Derivative of ATCC # BAA-535
*ΔmmpL7 M.marinum* M strain transformed with *pmsp12::tdTomato*	Cambier et al., 2014b	Derivative of ATCC # BAA-535
*Δerp M. marinum* M strain transformed with *pmsp12::tdTomato*	Takaki et al., 2013	Derivative of ATCC # BAA-535
*marP::Tn M. marinum* M strain transformed with *pmsp12::tdTomato*	Levitte et al., 2016	Derivative of ATCC # BAA-535
*ΔesxA M. marinum* M strain transformed with *pmsp12::tdTomato*	Osman et al., 2022	Derivative of ATCC # BAA-535
*ΔesxA M. marinum* M strain transformed with *::esxA*^WT^	Osman et al., 2022	Derivative of ATCC # BAA-535
*ΔesxA M. marinum* M strain transformed with *::esxA*^M83I^	Osman et al., 2022	Derivative of ATCC # BAA-535
*ΔesxA M. marinum* M strain transformed with *::esxA*^M93T^	Osman et al., 2022	Derivative of ATCC # BAA-535
*M. tuberculosis ΔleuDΔpanCD* mc^2^ 6206 transformed with *pmsp12::tdTomato*	Roca et al., 2019	N/A

**Chemicals, peptides, and recombinant proteins**		

BD Difco Middlebrook 7H9 broth (dehydrated)	Fisher Scientific	Cat# DF0713-17-9
Middlebrook 7H10 agar base	Sigma-Aldrich	Cat# M0303
L-leucine	Sigma-Aldrich	Cat# L8000; CAS: 61-90-5
Calcium pantothenate	Sigma-Aldrich	Cat# C8731; CAS: 137-08-6
Remel OADC Enrichment	Fisher Scientific	Cat# 11903262
RPMI 1640 medium	Sigma-Aldrich	Cat# R7509
Gibco Phenol-free RPMI	Thermo-Fisher	Cat# 11835030
XF DMEM, pH 7.4	Agilent	Cat# 103575-100
Instant Ocean Salt	ZM Systems	N/A
PTU (1-phenyl-2-thiourea)	Sigma-Aldrich	Cat# P7629; CAS: 103-85-5
Tango Buffer (10x)	Thermo Fisher	Cat# BY5
Phenol Red Sodium Salt	Sigma-Aldrich	Cat# P5530; CAS: 34487-61-1
Pronase	Sigma-Aldrich	Cat# P5147; CAS:9036-06-0
Tricaine (ethyl 3-amonobenzoate, methanesulfonic acid salt)	Fisher Scientific	Cat# 10743661; CAS: 886-86-2
TopVision Low Melt Agarose	Thermo Fisher	Cat# R0801
Rapamycin	Sigma-Aldrich	Cat# R0395; CAS: 53123-88-9
Torin1	Cambridge Bioscience	Cat# CAY10997; CAS: 1222998-36-8
Nifedipine	Cambridge Bioscience	Cat# N3228; CAS: 21829-25-4
Diltiazem HCl	Cambridge Bioscience	Cat# D3447; CAS: 33286-22-5
2DG (2-deoxy-D-glucose)	Sigma-Aldrich	Cat# D8375; CAS: 154-17-6
UK5099	Cambridge Bioscience	Cat# B1952; CAS: 56396-35-1
Digitonin	Acros	Cat# 407565000 CAS: 11024-24-1
Prazosin HCl	Sigma-Aldrich	Cat#: P7791; CAS: 19237-84-4
PMA (Phorbol 12-myristate 13-acetate)	Sigma-Aldrich	Cat#: P1585; CAS: 16561-29-8
Accutase	Sigma-Aldrich	Cat#: A6964
Paraformaldehyde, 16% w/v	Alfa Aesar	Cat# 11490570
Acridine Orange (2% solution in H_2_0)	Sigma-Aldrich	Cat# A9231; CAS: 65-61-2
MitoTracker Red CM-H_2_Xros	Thermo Fisher	Cat# M7513
eBioscience Fixable Viability Dye eFluor 660	Thermo Fisher	Cat# 65-0864-14
Tetramethylrhodamine, ethyl ester	Abcam	Cat# ab113852
SYTOX Green Nucleic Acid Stain	Thermo Fisher	Cat# S7020
AMPure XP beads for PCR Purification	Beckman Coulter	Cat# A63881
Precision Melt Supermix	BioRad	Cat# 172-5112
KASP V4.0 2X Master Mix	LGC Biosearch	Cat# KBS-1016-002
Mammalian Cell Lysis Buffer 5x	Abcam	Cat# ab179835
Alt-R Sp Cas9 Nuclease V3	IDT	1081058

**Critical commercial assays**		

Gibson Assembly Cloning Kit	New England BioLabs	Cat# E5510S
mMessage mMachine T7 Transcription Kit	Thermo Fisher	Cat# AM1344
Seahorse XFp Glycolytic Rate Assay Kit	Agilent	Cat# 103346-100
Seahorse XFp Mito Stress Test Kit	Agilent	Cat#103015-100
ATP Assay Kit (Colorimetric/Fluorometric)	Abcam	Cat# ab83355
Glucose-6-Phosphate Dehydrogenase Activity Assay Kit (Fluorometric)	Abcam	Cat# ab176722

**Experimental models: Cell lines**		

THP-1	ATCC	Cat# TIB-202, RRID:CVCL_0006

**Experimental models: Organisms/strains**		

Zebrafish (*Danio rerio*): wild type AB strain	University of Cambridge	ZDB-GENO-960809-7
Zebrafish: TL strain	University of Cambridge	ZDB-GENO-990623-2
Zebrafish: WIK strain	University of Washington	ZDB-GENO-010531-2
Zebrafish: *Tg(mpeg1:YFP)*^*w200*^	Roca and Ramakrishnan, 2013	ZDB-ALT-130130-3
Zebrafish: *Tg(mpeg1:Brainbow)*^*w201*^	Pagán et al., 2015	ZDB-ALT-150512-3
Zebrafish: *Tg(mfap4:tdTomato-CAAX)*^*xt6*^	Walton et al., 2015	ZDB-ALT-160122-3
Zebrafish: *Tg(ubib:secA5-YFP)*^*cu34*^	This work	N/A
Zebrafish: *Tg(CMV:EGFP-map1lc3b)*^*zf155*^	He et al., 2009	ZDB-ALT-091029-2
Zebrafish: *Tg(lysC:EGFP)*^*nz117*^	Hall et al., 2007	ZDB-ALT-071109-2
Zebrafish: *Tg(cd41:GFP)*	Lin et al., 2005	N/A
Zebrafish: *pycard*^*w216*^	Matty et al., 2019	ZDB-ALT-191009-1
Zebrafish: *mtor*^*fh178*^	This work	N/A
Zebrafish: *mtor*^*sa16755*^	Wellcome Trust Sanger Institute	ZDB-ALT-131217-12934
Zebrafish: *rptor*^*sa11537*^	Wellcome Trust Sanger Institute	ZDB-ALT-130530-177
Zebrafish: *rictora*^*sa15967*^	Wellcome Trust Sanger Institute	ZDB-ALT-130411-4494
Zebrafish: *rictorb*^*sa18403*^	Wellcome Trust Sanger Institute	ZDB-ALT-131217-14421
Zebrafish: *atg12*^*sa42684*^	Wellcome Trust Sanger Institute	ZDB-ALT-160601-8392
Zebrafish: *casp9*^*sa11164*^	Wellcome Trust Sanger Institute	ZDB-ALT-130411-1023
Zebrafish: *sting1*^*sa35634*^	Wellcome Trust Sanger Institute	ZDB-ALT-160601-4021
Zebrafish: *g6pd*^*sa24272*^	Wellcome Trust Sanger Institute	ZDB-ALT-161003-11894

**Oligonucleotides**		

*mtor*^*fh178*^ forward primer for genotyping by HRMA 5’-TCACAGTATCAGATCTTCATTCCTATGGT-3’	This paper	N/A
*mtor*^*fh178*^ reverse primer for genotyping by HRMA 5’-ACATCATAGCGCTGGTGATTGAT-3’	This work	N/A
*mtor*^*sa16755*^ forward primer for genotyping by HRMA 5’-TGACTACAGCACCAGCGAGA-3’	This work	N/A
*mtor*^*sa16755*^ reverse primer for genotyping by HRMA 5’-ATGGTGTGGTGATTGGACAG-3’	This work	N/A
Alt-R crRNA Dr.Cas9.NDUFAF1.1.AA 5’-TGGAACAGACCCGTGTCGTG-3’	IDT	N/A
Alt-R crRNA Dr.Cas9.NDUFAF1.1.AB 5’-TATCGAGTCTCTCCATCACG-3’	IDT	N/A
Alt-R crRNA Dr.Cas9.NDUFAF1.1.AC 5’-GGTCCCATACAGCAAACACG-3’	IDT	N/A
Alt-R crRNA Dr.Cas9.NDUFAF1.1.AD 5’-CGTGTGTCTGGAGGCTGACC-3’	IDT	N/A
Alt-R CRISPR-Cas9 tracrRNA	IDT	1073191
Forward primer for genotyping *ndufaf1* AA mutagenesis by HRMA 5’-AGAGCACATGCTGGAACAGA-3’	This work	N/A
Reverse primer for genotyping *ndufaf1* AA mutagenesis by HRMA 5’-TGTTTTTGCCCAGACTGACA-3’	This work	N/A
Forward primer for genotyping *ndufaf1* AB, AC mutagenesis by HRMA 5’-CGCAGTGTGGCTTATGTCAG-3’	This work	N/A
Reverse primer for genotyping *ndufaf1* AB, AC mutagenesis by HRMA 5’-TTGGAGCGCATAGAGCAGTA-3’	This work	N/A
Forward primer for genotyping *ndufaf1* AD mutagenesis by HRMA 5’-AGGAGCAAGTTTGAGCGAGA-3’	This work	N/A
Reverse primer for genotyping *ndufaf1* AD mutagenesis by HRMA 5’-AAGCCAAAGTGCTTCCTGAC-3’	This work	N/A
pDestTol2pA2_ubi:EGFP forward primer for vector fragment amplification for Gibson Assembly 5’-GGCGGTGGAAGATCTGGG-3’	This work	N/A
pDestTol2pA2_ubi:EGFP reverse primer for vector fragment amplification for Gibson Assembly 5’-GGTCCAGCCTGCTTTTTTG-3’	This work	N/A
secA5-YFP forward primer for insert fragment amplification for Gibson Assembly 5’-aaagcaggctggaccATGCATAAGGTTTTGCTG-3’	This work	N/A
secA5-YFP reverse primer for insert fragment amplification for Gibson Assembly 5’-agatcttccaccgccGATGAATTAATTCGAGCTCC-3’	This work	N/A
ubb:secA5 joint sequence forward primer to validate assembled product 5’-TCGTTTAACATGGGAGAAGTGC-3’	This work	N/A
ubb:secA5 joint sequence reverse primer to validate assembled product 5’-AGCCTTTCATAGCCTTCCGA-3’	This work	N/A
YFP-SV40pA joint sequence forward primer to validate assembled product 5’-CTGTACAAGTAAAGCGGCCG-3’	This work	N/A
YFP-SV40pA joint sequence reverse primer to validate assembled product 5’-GTAAAACGACGGCCAGTGAA-3’	This work	N/A

**Recombinant DNA**		

T7-TPase	Khattak et al., 2014	RRID:Addgene_51818
pDestTol2pA2_ubi:EGFP	Mosimann et al., 2011	RRID:Addgene_27323
pBH-UAS-secA5-YFP	van Ham et al., 2010	RRID:Addgene_32359
pTol2-ubb:secA5-YFP	This work	N/A

**Software and algorithms**		

NIS Elements (5.21)	Nikon	N/A
IMARIS (8.2) and IMARIS for Cell Biologists (9.1)	Bitplane	N/A
FlowJo 10	TreeStar	N/A
Prism (versions 7 and 9)	GraphPad	N/A
ImageJ	https://imagej.nih.gov/ij/	N/A
Fluorescent Pixel Count Macro (Image J)	Takaki et al., 2013	N/A
AssayR	Wills et al., 2017	N/A
MetaboAnalyst 5.0	Pang et al., 2021	N/A
Photoshop CS6	Adobe	N/A
Illustrator CS6	Adobe	N/A

### Resource availability

#### Lead contact

Additional information and requests for resources and reagents should be directed to and will be fulfilled by the lead contact, Lalita Ramakrishnan (lalitar@mrc-lmb.cam.ac.uk).

#### Materials availability

Plasmids and zebrafish lines generated in this study are available from the lead contact.

#### Data and code availability

• Microscopy and flow cytometry data reported in this paper will be shared by the lead contact upon request. The unprocessed metabolomics data generated in this study are available from the lead contact.
• This paper does not report original code.
• Any additional information needed to reanalyze the data reported in this paper is available from the lead contact upon request.

### Experimental model and subject details

#### Ethics statement

Zebrafish husbandry and experiments were carried out in compliance with guidelines from the UK Home Office and the US Public Health Service Policy on Human Care and Use of Laboratory Larvae using protocols approved by the Animal Welfare and Ethical Review Body of the University of Cambridge and the Institutional Animal Care and Use Committee of the University of Washington, respectively.

#### Zebrafish husbandry and infections

Zebrafish embryos and larvae (of undetermined sex due to their early stages of development) of the wild-type AB strain (Zebrafish International Resource Center, ZIRC), TL strain (ZIRC), or of mixed AB/TL backgrounds were used in experiments. The *Tg(mpeg1:YFP)*^*w200*^*, Tg(mpeg1:Brainbow)*^*w201*^ (described as *mpeg1:tdTomato*), *Tg(mfap4:tdTomato-CAAX)*^*xt6*^*, Tg(CMV:EGFP-map1lc3b)*^*zf155*^, *Tg(lysC:EGFP)*^*nz117*^ *, Tg(cd41:GFP)*, and *Tg(ubb:secA5-YFP)*^*cu34*^ (this work) fluorescent reporter lines were maintained in the AB strain (Hall et al., 2007; He et al., 2009; Lin et al., 2005; Pagán et al., 2015; Roca and Ramakrishnan, 2013; Walton et al., 2015). The *pycard*^*w216*^ mutant line (Matty et al., 2019) and *mtor*^*fh178*^ mutant line (this work) were generated and maintained in the AB strain. The *mtor*^*sa16755*^, *rptor*^*sa11537*^, *rictora*^*sa15967*^, *rictorb*^*sa18403*^, *atg12*^*sa42684*^, *casp9*^*sa11164*^, *sting1*^*sa35634*^, and *g6pd*^*sa24272*^ mutant lines (Wellcome Trust Sanger Institute) (Kettleborough et al., 2013) were generated in the TL strain and maintained in either the TL strain (*rictora*^*sa15967*^, *rictorb*^*sa18403*^, and *g6pd*^*sa24272*^) or mixed AB/TL backgrounds (*mtor*^*sa16755*^, *rptor*^*sa11537*^, and *atg12*^*sa42684*^, *casp9*^*sa11164*^, and *sting1*^*sa35634*^). Zebrafish of the WIK strain (ZIRC) were used to map *mtor*^*fh178*^. Zebrafish were maintained in buffered reverse osmotic water systems under a 14-hr light/10-hr dark cycle. Zebrafish larvae were fed paramecia twice daily, while juvenile and adult zebrafish were fed at least twice a day with dry food and brine shrimp. Zebrafish embryos were collected and cultured in reverse osmosis water containing 0.18g/L Instant Ocean Salt supplemented with 0.25μg/mL methylene blue at 28.5°C. On 1 dpf, embryos to be used in experiments were transferred to 0.5x E2 medium (7.5mM NaCl, 0.25mM KCl, 0.5mM MgSO_4_, 0.075mM KH_2_PO_4_, 0.025mM Na_2_HPO_4_, 0.5mM CaCl_2_, and 0.35mM NaHCO_3_) supplemented with 0.003% PTU (1-phenyl-2-thiourea, Sigma) to inhibit melanin synthesis.

For infections, 2 dpf larvae of undetermined sex (due to early developmental stage) were dechorionated manually or with ≤0.5mg/mL pronase (Sigma-Aldrich) and then anesthetized with fish water containing 0.025% tricaine (Sigma). Larvae were injected via the caudal vein or the hindbrain ventricle using single-cell suspensions of Mm of known titer to deliver ∼100 - 250 bacteria per 3 – 5 nL injection as previously described (Takaki et al., 2013). Phenol red sodium salt (≤ 1% w/v diluted in PBS, Sigma-Aldrich) was used as an injection tracer. Inoculums were confirmed by injecting onto Middlebrook 7H10 agar plates (supplemented with oleic acid, albumin, dextrose, and Tween-80 plus hygromycin B or kanamycin, as appropriate. For experiments with mutant lines, wild-type and heterozygous siblings were used as comparators, animals were genotyped after data acquisition. In experiments involving drug treatments, infected animals were randomly assigned to treatment groups. Unless indicated, treatments were initiated immediately after infection via soaking, and drug exposure was maintained until the experimental endpoint. UK5099 (Cambridge Bioscience) was changed daily.

#### THP-1 macrophage culture and infections

Monocytic human THP-1 cells (ATCC) were grown at 37°C, 5% CO_2_ in RPMI medium (Sigma-Aldrich) supplemented with 10% FBS (Gibco), L-glutamine (Gibco), Penicillin, and Streptomycin (complete RPMI). To differentiate THP-1 cells into macrophages, cells were seeded at 5 x 10^5^ cells/mL in flat-bottomed tissue culture plates (2.5 x 10^5^ cells/well of 24-well plate) and stimulated with 100nM PMA Phorbol 12-myristate 13-acetate (Sigma-Aldrich) for two days. The resulting adherent cells were then washed with complete RPMI and rested for two days prior to infection. For microscopy experiments, cells were plated on optical bottom plates (Perkin Elmer or MatTek). Cells were pre-incubated overnight with pharmacological compounds or matching concentrations of vehicle (≤0.5% DMSO). On the infection day, cells were washed with antibiotic-free complete RPMI and infected with single-cell suspensions of Mm or Mtb for five hours in a 33°C, 5% CO_2_ incubator (for Mm) and 37°C, 5% CO_2_ incubator (for Mtb). After infection, cells were washed with antibiotic-free complete RPMI supplemented with the corresponding pharmacological compounds and returned to the appropriate incubators. For experiments with the leucine and pantothenate double auxotroph *ΔleuDΔpanCD* Mtb, complete RMPI was also supplemented with 0.05 mg/mL L-leucine and 0.024 mg/mL calcium pantothenate (Sigma-Aldrich). Multiplicity of infection was determined by calculating the number of mycobacterial colony forming units (CFU) per cells plated in each well.

### Method details

#### Bacterial strains

*M. marinum* M strain (ATCC #BAA-535) and its mutant derivatives *ΔESX-1*, *ΔmmpL7*, *Δerp*, *marP::tn*, and *ΔesxA* expressing BFP2, mWasabi, tdTomato, or tdKatushka2 under the control of the *msp12* promoter (Cambier et al., 2014b; Cosma et al., 2006; Levitte et al., 2016; Osman et al., 2022; Takaki et al., 2013) were grown at 33°C under hygromycin B (Cambridge Bioscience) or kanamycin (Sigma-Aldrich) selection in Middlebrook 7H9 medium (BD Difco) supplemented with oleic acid, albumin, dextrose, and Tween-80 (Sigma-Aldrich) (Takaki et al., 2013). *M. tuberculosis ΔleuDΔpanCD* mc^2^ 6206 expressing *msp12:tdTomato* was grown at 37°C under hygromycin B and kanamycin selection in Middlebrook 7H9 medium supplemented with oleic acid, albumin, dextrose, Tween-80, catalase, and 0.05 mg/mL L-leucine and 0.024 mg/mL calcium pantothenate (Sigma-Aldrich) (Roca et al., 2019; Sampson et al., 2011).

#### Zebrafish genotyping

DNA from adult fin clips or whole larvae was extracted using the HotSHOT method (Truett et al., 2000). Animals were genotyped by High Resolution Melt Analysis (HRMA) of PCR products (Garritano et al., 2009) or by Kompetitive Allele-Specific PCR (KASP) assay (LGC Biosearch) (He et al., 2014) in a CFX Connect thermocycler (BioRad). DNA from 2DG-treated animals was purified with AMPure XP beads (Beckman Coulter) according to the manufacturer’s instructions prior to PCR.

#### Mapping of *mtor*^*fh178*^

Zebrafish carrying the *fh178* allele were outcrossed to the WIK strain for mapping as previously described (Tobin et al., 2010). *fh178* was mapped to chromosome 8 initially between the markers z7370 and z14670. Further mapping defined a critical two-gene interval that included *mtor* and *qars1* to the right of a single nucleotide polymorphism in the 3’ end of *angptl7* (1 recombination in 302 meioses) and to the left of a polymorphism in intron 2 of *ogg1* (2 recombinations in 300 meioses). Sequencing of cDNAs isolated from mutants and wild-type animals in the critical region identified a stop codon in exon 24 of *mtor* in the mutant but not wild-type animals. This mutation segregated absolutely with the *fh178* mutants (no recombinants).

#### Creation of *Tg(ubb:secA5-YFP)*^*cu34*^

The Tol2 *ubb:secA5-YFP* plasmid was assembled from PCR-amplified fragments of pDestTol2pA2_ubi:EGFP (RRID: Addgene 27323) (Mosimann et al., 2011) and pBH-UAS-secA5-YFP (RRID: Addgene 32359) (van Ham et al., 2010) the Gibson Cloning kit (New England Biolabs). Correct plasmid assembly was confirmed by diagnostic PCR of joined segments and by Sanger sequencing. Tol2 transposase (RRID: Addgene 51818) (Khattak et al., 2014) was in vitro transcribed with the T7 mMessage/mMachine kit (Thermo Fisher) according to the manufacturer’s instructions. Tol2 *ubb:secA5-YFP* plasmid and transposase mRNA were co-injected into one-cell embryos of the wild-type AB strain as previously described (Suster et al., 2011). G0 larvae expressing the transgene were identified by fluorescence microscopy and raised to adulthood. Potential founders were identified through pairwise crosses of G0 adults and non-transgenic AB fish. A single F1 transgenic animal was used to establish the line.

#### CRISPR-Cas9 mutagenesis

The mutagenesis procedure was adapted from published methods (Burger et al., 2016; Wu et al., 2018). Guide RNAs (60 μM) were generated by complexing equimolar amounts of Alt-R tracrRNA and individual *ndufaf1*-specific Alt-R crRNAs (Dr.Cas9.NDUFAF1.1.AA, AB, AC, and AD; IDT) in nuclease-free Duplex Buffer (IDT) at 95°C for 5 minutes. Aliquots of complexed RNA were stored at -20°C. To produce ribonucleoprotein complexes (RNPs) (10 μM), guide RNA pools were combined with Alt-R Sp Cas9 Nuclease V3 (IDT) at an equimolar ratio of total RNA to Cas9 in working buffer (20 mM HEPES, 150 mM KCl, pH 7.5) and incubated at 37°C for 10 minutes. Wild-type zebrafish of the AB strain were injected at the 1-cell stage with ∼2 nL of RNP to create G0 crispants or ∼2nl of working buffer to produce unmutagenized experimental control animals. Mutagenesis was assessed in individual animals by HRMA of PCR amplicon spanning each of the CRISPR targets.

#### Parabiosis

Parabiosis of zebrafish embryos was performed as previously described (Demy et al., 2013). Briefly, blastulae derived from synchronized *mtor*^*fh178/+*^*; Tg(mpeg1:YFP)*^*w200*^ and *Tg(mpeg1:tdTomato)* incrosses were manually dechorionated, paired, scratched with an aluminum silicate microinjection needle, and allowed to fuse in high-calcium Ringer’s solution (116 mM NaCl, 2.9 mM KCl, 10 mM CaCl_2_, 5 mM HEPES, pH 7.2) supplemented with 50 U/mL penicillin and 50 U/mL streptomycin. Only parabiotic pairs fused at the head, devoid of gross morphological abnormalities, and sharing blood circulation were used in experiments. On 2 dpf, each parabiotic animal was infected via the caudal vein to ensure similar distribution of mycobacteria between the pair’s caudal hematopoietic tissues. The bodies of each parabiotic pair were micro-dissected and individually genotyped after completion of the experiment.

#### Microscopy and image analyses

Widefield fluorescence microscopy was performed as previously described (Takaki et al., 2013). Bacterial burdens were determined with a Nikon Eclipse Ti-E inverted microscope using a 4x objective and running a fluorescent pixel counts macro in ImageJ (National Institutes of Health) (Takaki et al., 2013). Macrophages were counted manually with a Nikon Eclipse E600 upright microscope using a 20x objective. For serial imaging of individual zebrafish, larvae were housed individually in 96-well plates under standard husbandry conditions.

Laser scanning confocal microscopy was performed as described (Pagán et al., 2015). Larvae were anesthetized in PTU-supplemented 0.5x E2 medium containing 0.025% tricaine and embedded in 2% low melt agarose (TopVision) on optical-bottom plates (MatTek). Imaging was performed with a Nikon A1R confocal microscope using 20x or 40x objectives and the galvano or resonant scanners. 0.9 - 2 μm optical sections were combined to generate 15 - 60 μm z stacks. For time-lapse microscopy of zebrafish larvae, imaging was done at 27°C using a microscope incubator (Okolab), using acquisition intervals of 2 - 5 minutes for 6 hours. For serial imaging of individual zebrafish, larvae were removed from the agarose using jeweler’s forceps and housed individually under standard husbandry conditions. For time-lapse microscopy of Mm-infected THP-1 cells, samples were imaged at 33°C, 5% CO_2_ in antibiotic-free, phenol-free complete RPMI containing 50 nM SYTOX Green nucleic acid stain (Invitrogen) for 4 hours.

The acquired images were processed using the denoising feature in the Elements software (Nikon). The surface rendering feature of Imaris (Bitplane) was used to quantify intramacrophage bacterial burdens and mROS levels; the number of acridine orange foci in the midbrain and LC3 puncta in neuromasts, clusters of mechanosensory hair cells of the lateral line in fish; phospho-S6 and total S6 MFIs, and the frequencies of galectin-8-associated Mm per imaging field (Thomas et al., 2015). The numbers of dying macrophages and macrophages with galectin-8-decorated Mm foci were counted manually.

#### Acridine orange staining

Zebrafish larvae were transferred to 1.5 mL microcentrifuge tubes and stained with 1 mL of 2.4μg/mL acridine orange in 0.5x E2 medium with PTU for 30 minutes with rotation and protected from light (Roca et al., 2019). Stained larvae were then rinsed in 0.5x E2 medium with PTU and imaged by confocal microscopy.

#### Mitochondrial vital dye staining

Zebrafish larvae were injected with ∼1 nL of 10 mM MitoTracker Red CMXH_2_-Xros via the caudal vein immediately prior to imaging by confocal microscopy (Roca and Ramakrishnan, 2013).

THP-1 macrophages were incubated with 40nM MitoTracker Deep Red (Thermo Fisher) and/or 100 nM tetramethylrhodamine, ethyl ester (TMRE) (Abcam) for 20 minutes in a 37°C, 5% CO_2_ incubator according to the manufacturers’ instructions. For flow cytometric analysis of TMRE, cells were then washed with complete RPMI media, stained with FVD eF660, and run without chemical fixation.

#### Immunostaining

The staining procedure was performed as previously described (Osman et al., 2020). THP-1 cells were fixed in PFA solution (4% w/v paraformaldehyde in PBS) at room temperature (RT) for at least 30 minutes. Cells were then washed twice with PBS and incubated in permeabilization/block (PB) solution (0.1% Triton-X 100, 1% bovine serum albumin in PBS) for 30 minutes at RT, and subsequently stained with Alexa Fluor 647-conjugated rabbit anti-phospho-S6^S235/S236^ or total S6 (Cell Signaling Technology) or goat anti-human galectin-8 antibody (R&D Systems) diluted in PB solution overnight at 4°C. The following day, cells stained with the galectin-8 antibody were washed three times with PBS and stained with Alexa Fluor 488-conjugated donkey anti-goat IgG (Thermo Fisher) in PB solution for one hour at RT. Cells were then washed three times with PBS and maintained in PBS for imaging by confocal microscopy. In some experiments, cells nuclei were stained with Hoechst 33342 (10μg/mL) for 20 minutes at RT prior to imaging.

#### Flow cytometry

THP-1 macrophages were stained with eBioscience Fixable Viability Dye (FVD) eFluor 660 (Thermo Fisher), detached with Accutase (Sigma-Aldrich), and transferred to polystyrene FACS tubes (Sarstedt). Single-cell suspensions were washed with MACS buffer (0.5% bovine serum albumin, 2mM EDTA in PBS, ph7.2) at 290 x g for 5 minutes at 4°C, and in most cases, fixed in 4% PFA solution overnight at 4°C. The following day, samples were washed with and resuspended in MACS buffer. Data were acquired in LSRII or Fortessa flow cytometers (BD Biosciences) and analyzed in FlowJo 10 (TreeStar), gating on single-cell events.

#### Cytochrome c release assay

The cytochrome c release assay was adapted from Lienard et al. (2020). 7 hours after infection with tdTomato-expressing Mm, cells were stained with eBioscience Fixable Viability Dye (FVD) eFluor 660 (Thermo Fisher), detached with Accutase (Sigma-Aldrich), transferred to polystyrene FACS tubes (Sarstedt), and washed first with ice-cold MACS buffer (0.5% bovine serum albumin, 2mM EDTA in PBS, ph7.2) and then ice-cold PBS by centrifugation at 290 x g for 5 minutes at 4°C. Cells were incubated in ice-cold permeabilization buffer (50 μg/mL digitonin, 100 mM KCl) for 5 minutes and immediately fixed in 4% PFA solution to stop the permeabilization reaction. Cells were then centrifuged as above and fixed overnight in 4% PFA solution at 4°C. Samples were subsequently permeabilized with eBioscience Permeabilization Buffer (Thermo Fisher) and stained with AlexaFluor 488 anti-cytochrome c antibody (BioLegend) for 2 hours at room temperature. Samples were analyzed by flow cytometry by gating on the viable, infected (FVD eFluor 660^-^ tdTomato^+^) single-cell events.

#### ATP quantification

ATP levels were quantified by fluorimetry in a CLARIOStar Plus (BMG Labtech) microplate reader using the ATP Assay Kit (Abcam) according to the manufacturer’s instructions. 10^6^ THP-1 macrophages were analyzed per replicate.

#### Measurement of G6PD activity

Individual 5 dpf larvae from an *g6pd*^*sa24272/+*^ incross were lysed using Mammalian Cell Lysis Buffer (Abcam). A small amount of each lysate was used to genotype the larvae. G6PD activity in individual larvae was determined by fluorimetry in a CLARIOStar Plus (BMG Labtech) microplate reader using the G6PD Activity Assay Kit (Abcam) according to the manufacturer’s instructions.

#### Metabolic analyses

For metabolite quantification, THP-1 cells (2 x 10^6^ cells/sample, four replicates/condition) were washed in ice-cold PBS, and metabolites were extracted with 150 μL extraction buffer (50:30:20, methanol:acetonitrile:water) cooled on dry ice for 30 minutes beforehand. Samples were then centrifuged at maximum speed for 10 minutes at °C to remove protein debris, and supernatants were stored at -80°C until acquisition. Liquid chromatography-mass spectrometry (LC-MS) was carried out using an Agilent 1290 Infinity II UHPLC in line with a Bruker Impact II QTOF operating in negative ion mode, as previously described (Edwards-Hicks et al., 2020). LC separation was performed on a Phenomenex Luna propylamine column (50 x 2 mm, 3 μm particles) using a solvent gradient of 100% buffer B (5 mM ammonium carbonate in 90% acetonitrile) to 90% buffer A (10 mM NH_4_ in water). Flow rate was from 1000 to 750 μL/min. Autosampler temperature was 5°C, and injection volume was 2 μL. Metabolites were quantified using AssayR (Wills et al., 2017). The hierarchical clustering heatmap was generated in MetaboAnalyst 5.0 using Euclidean distance measure, the Ward clustering algorithm, and autoscale feature standardization (Pang et al., 2021).

Metabolic flux measurements were carried out with the Seahorse XFp Glycolytic Rate Assay and Cell Mito Stress Test kits (Agilent) according to the manufacturer’s instructions. On the day of the assay, 4 x 10^4^ differentiated THP-1 macrophages were seeded per well of the XFp cartridge, allowed to adhere for 2 hours in a 37°C, 5% CO_2_ incubator, and then incubated with assay medium (XF DMEM medium with 10 mM glucose and 2 mM glutamine but without sodium pyruvate (Agilent)) for 45 − 60 minutes in a non- CO_2_ 37°C incubator prior to data acquisition in a Seahorse XFp analyzer (Agilent).

### Quantification and statistical analyses

Statistical analyses were performed on Prism (GraphPad) with each statistical test, definitions of center (mean or median) and dispersion (standard error of the mean), and the number of samples per group (n, referring to animals, individual cells, or pools of cells in a tissue culture well) indicated in the corresponding figure legends and panels. The unpaired, two-tailed Student’s *t* test was used to determine statistical significance between the mean values of two groups. One- or two-way Analysis of Variance (ANOVA) with Tukey’s or Sidak’s post tests were used to compare the mean values of three or more groups. Fisher’s exact test was used to analyze contingency table data (i.e., number of animals with mycobacterial cording vs. no cording). Statistical significance was determined by *P* < 0.05. Statistically significant comparisons in each figure are indicated with asterisks, ^∗^*P* < 0.05; ^∗∗^*P* < 0.01; *^∗∗∗^P* < 0.001; *^∗∗∗∗^P* < 0.0001.

## Supplemental information

Table S1. Metabolic profiles of uninfected and Mm-infected THP-1 macrophages treated with Torin1, 2DG, or DMSO, related to Figures 5 and S3
