# Supplementary material for: mTOR-regulated mitochondrial metabolism limits mycobacterium-induced cytotoxicity
Source: Cell. 2022 Sep 29;185(20):3720–3738.e13. doi: 10.1016/j.cell.2022.08.018 (PMC9596383; doi:10.1016/j.cell.2022.08.018)
Supplement: Table S1. Metabolic profiles of uninfected and Mm-infected THP-1 macrophages treated with Torin1, 2DG, or DMSO, related to Figures 5 and S3 [file mmc1.pdf]

Cell, Volume *185*

Supplemental information

mTOR-regulated mitochondrial metabolism limits mycobacterium-induced cytotoxicity

Antonio J. Pagán, Lauren J. Lee, Joy Edwards-Hicks, Cecilia B. Moens, David M. Tobin, Elisabeth M. Busch-Nentwich, Erika L. Pearce, and Lalita Ramakrishnan

Table S1. Metabolic Profiles of Uninfected and Mm-infected THP-1 Macrophages Treated with Torin1, 2DG, or DMSO, Related to Figure 5 and Figure S3.

	Normalized Counts Per Biological Replicate.																							
Metabolites	n01 UN DMSO	n02 UN DMSO	n03 UN DMSO	n04 UN DMSO	n05 UN Torin 1	n06 UN Torin 1	n07 UN Torin 1	n08 UN Torin 1	n09 UN 2DG	n10 UN 2DG	n11 UN 2DG	n12 UN 2DG	n13 Mm DMSO	n14 Mm DMSO	n15 Mm DMSO	n16 Mm DMSO	n17 Mm Torin1	n18 Mm Torin1	n19 Mm Torin1	n20 Mm Torin1	n21 Mm 2DG	n22 Mm 2DG	n23 Mm 2DG	n24 Mm 2DG
Acetyl CoA	52491	30385	38099	40894	22501	21678	27794	29051	24868	26499	27482	32089	29255	31323	36401	19537	21318	17097	15824	22298	33682	27606	24343	30240
Aconitic Acid	1365926	1066801	1168854	1121209	164720	198692	185748	223063	358255	467436	384625	446899	921138	1088699	1138256	759516	257359	302472	290579	371092	486800	412393	393118	564892
ADP	1345310	605373	842051	642504	597317	643032	725953	774759	518303	738896	739796	905147	661895	609531	833873	594090	325517	255169	287870	268836	599148	487781	510803	555942
AMP	641560	186613	318324	160147	347877	312646	473996	348814	209674	330418	332269	366911	278462	163785	301333	282974	133038	73133	86245	72840	225889	169845	193303	197510
Arginine	649868	755097	662466	739952	569815	672042	664626	688534	628624	697860	635388	599645	463597	454960	463458	347679	332130	385117	404983	420951	439693	507445	530867	727001
Argininosuccinic Acid	7093	4878	11259	6092	0	0	0	0	0	0	2598	1832	7847	11152	11402	2973	0	0	0	0	4320	0	0	6134
Asparagine	4067125	3321188	3229438	3261043	2972013	3092156	3142511	3346368	2334617	3164841	2603801	3092999	2923208	2878899	3438850	2272626	1487272	1276696	1363581	1336634	2574233	2390281	2430101	3105632
Aspartic Acid	6697457	4165832	5171314	4475326	879255	995256	1145266	1243230	1742045	2258211	1844763	2395504	4349900	5139298	6229693	3424719	841818	765794	890780	893047	1977825	1462858	1444019	1703390
ATP	1113271	335999	770413	875623	195911	226564	379108	319638	464633	256557	301168	611807	1249801	1134884	550969	318621	345311	235658	375576	569830	1015546	766772	310305	387429
Biotin	12897	13457	18982	8935	13261	9904	17898	6837	12184	5415	6692	9606	19250	13333	23633	14449	5769	5725	9493	7586	3301	3707	9700	0
cAMP	1288609	307072	559915	151356	363248	309212	504514	352184	176748	466103	446456	504477	156184	70093	162686	158343	27793	28982	27722	24894	71266	55001	85425	47329
CDP	52442	22821	28830	24977	56504	55975	65839	65038	23194	28114	26508	37331	22703	21379	24785	23891	35424	25221	36500	26973	21602	17665	19471	21715
Citric Acid	35180074	27684255	30517541	31006344	7917489	9476379	8047761	10666761	13560053	16890978	14991753	16395142	25417778	31517297	30477625	21672612	8965036	10629868	11589476	13738032	19345409	16479324	14225520	20057446
CMP	80197	37994	55628	46689	48031	54827	72170	67093	41319	54743	51448	71638	36556	43297	43382	22870	25750	26026	25311	30393	67194	46941	38790	54681
CoenzymeA	15228	9161	13308	12524	7747	8889	8532	5565	11469	8668	10907	10924	11559	10723	11403	8748	7991	6209	8256	7273	12500	11176	10278	10335
CTP	69121	18244	49024	52875	24375	31236	50427	49999	31968	16566	19380	44640	80154	71312	34257	17398	73582	53809	96381	138308	69696	55267	17333	25849
Erythrose-4-phosphate	16439	2817	4550	0	0	0	0	0	39837	55307	43318	40547	0	0	0	0	0	0	0	0	58485	36430	41111	40311
FAD	97398	71821	85351	71567	44391	43652	59789	58753	60186	72569	70443	76248	68611	66837	92946	62977	39941	40619	40043	38230	74567	61763	71699	72621
Folic Acid	7497	7345	7972	10466	424	8521	6870	15370	6681	9122	7564	6461	0	8271	3233	1279	4672	1855	8807	12008	11652	8762	8838	29178
Fructose-1,6-bisphosphate	31326	13554	29479	28274	14461	11799	25540	16572	12482	11168	9177	16449	23142	25621	21165	8950	23457	22178	28225	37609	18947	18652	10499	14925
Fumaric Acid	1605901	1246802	1396122	1312802	768150	751692	808149	848563	937674	1081708	1000311	997476	1361699	1531572	1515359	1137145	692795	743090	712828	854976	1065411	917056	895473	1060763
GDP	59734	15094	33650	28789	22459	20686	34480	24844	28463	28295	29562	50614	32892	25275	29700	22744	18261	12373	13844	12889	36900	30804	21545	24116
Glucose	184720	135539	144804	151158	94533	81441	91451	93740	39010824	42692289	39338172	41989983	139813	146726	141761	126635	69398	68598	71921	101814	35966614	30928260	33624793	34442668
Glucose-6-phosphate	3071821	2081789	2457768	2390771	894608	965799	1258652	1144202	1121845	1335857	1257922	1419597	1707834	1776537	2118072	1257570	510083	561794	618915	666001	1508856	1262499	1204491	1426724
Glucose-6-phosphate Peak2	123119	124350	98656	99205	67462	90931	66515	100064	5532689	6629624	6341225	6811482	84672	111946	89902	68670	39482	44307	77571	47646	6928282	6181670	6452547	6401583
Glutamic Acid	30105918	23098356	26127293	23674515	13497861	16508037	18957695	19867578	12316755	15350090	13889308	14441351	20816778	20973679	24017283	17309295	9125680	9305947	10570804	10925876	14533800	10880933	9167533	13416859
Glutamine	6910801	6675434	6390529	7181325	4033138	4572300	5003811	4612674	4160699	5560552	4458007	5180935	5268127	5559010	5318208	3067465	1555223	1724759	1815836	2153905	4419534	4363130	3948767	6187370
Glycerol phosphate	4489539	3870426	3969160	3591272	2880003	3123120	3194484	3447684	3485460	3946086	3740399	3921140	3484465	3281221	3946163	3206830	3211940	2784212	2895870	2764461	3732257	3385108	3480522	3695836
GMP	12717	6561	5632	8500	10978	6716	14392	8252	11062	10139	11960	12336	8230	4007	6993	6936	5986	3644	5067	4618	9107	10189	10287	11873
GSH	51021305	39195607	42318292	38168290	18070110	21189790	23801826	26039607	27548050	35157557	31814268	34954517	35238978	38493945	45655005	27993357	15300343	13387809	15464908	15742196	38481047	28664774	30087268	36620555
GSSG	3796772	2958720	3669246	3536803	2220788	1744730	2249447	2064274	2645831	2876438	2486541	2946976	3835303	3366855	4228071	2904111	2046274	1689338	1668389	1557847	3015814	2916438	3137044	2873379
GTP	14638	8902	9171	10879	4997	7262	6682	4752	9398	9953	9803	9785	14442	11567	11941	10263	6186	6479	5791	5887	16158	13693	14931	8735
Histidine	1082979	898075	897599	1001855	755017	925615	910887	1050898	904113	947875	925535	1103998	417371	466782	447177	286854	385826	299283	376652	413967	706912	674447	549693	761079
Hydroxyglutaric Acid	1605146	1053512	1356231	1185585	528484	547980	640492	632798	772712	883387	733714	831545	1069880	1167980	1348838	850680	457943	453728	496082	540522	866779	643726	751541	808587
Hypoxanthine	593300	548682	783568	471133	525270	366046	624698	418723	393186	453195	346842	325205	618113	390793	632754	451169	456638	379307	294840	265797	208028	322075	382416	263989
IMP	65524	48530	89636	56487	139350	68172	171692	59690	96559	129557	99057	97157	147262	79266	146764	116747	82884	74321	69911	47965	122114	135861	176136	92068
Itaconic Acid	433880	415357	486241	443682	401569	387045	434185	428319	450267	426129	426188	402279	500134	598792	395362	415112	326586	439538	474982	520563	472106	442492	433036	420801
Lysine	18030	25144	20124	16273	20587	19821	16854	12022	20264	26024	20959	18469	10960	4049	12579	8372	2249	10904	10117	6356	6858	11742	18648	16742
Malic Acid	13900229	9754925	11142769	10442481	3598439	3846706	4235548	4650461	5977579	7625603	6058228	6994597	10556580	12199093	12290554	8146952	3157056	3355548	3537423	4203536	7222512	5688630	5505901	7363775
Methionine	6767	3559	14770	6084	4449	2878	11604	4631	9465	3771	3854	0	10623	0	0	6226	4235	4544	509	2439	0	3197	4430	3366
NAD	19991	12426	18661	19633	10328	9180	13950	12026	16295	17680	15176	13827	11280	11991	12265	10451	9399	6671	5890	6674	13921	12342	11835	12721
NADH	132393	81257	83175	85010	72838	69619	89628	100057	72381	93738	85686	93702	69058	78574	83421	58646	48693	45112	51692	46970	99535	76733	84673	119763
NADP	26551	20491	23257	19172	10496	10671	13369	15398	9822	16220	17326	13976	19206	19041	25666	17840	10232	8949	8969	9945	15890	13171	13314	14852
NADPH	22887	11478	17474	20764	7551	9169	14930	11824	8752	6317	6269	11672	20451	24480	23745	11577	13594	10417	13994	13757	16851	13093	9936	15564
Nicotinic Acid	1059335	837720	893783	886559	1359764	1602453	1498501	1630017	596001	595156	492109	656583	1717812	1719728	1573784	1358121	1662734	1848390	1921691	2057986	836291	801940	692215	787793
O-phosphoserine	33020	42491	24322	25571	29758	31951	27529	30723	16213	31642	27431	20582	20050	27475	24960	35480	16652	18929	22978	17372	28587	11403	23923	27528
Oxaloacetic Acid	21929	17702	17208	22503	2473	7699	1958	7213	9349	10010	10754	9420	17505	18293	18531	13793	6806	7229	5799	9750	4047	10403	7522	14165
Palmitic Acid	693772	592521	555557	553255	553367	638377	531160	569843	604838	519230	593015	679691	739144	610178	649487	561751	532477	597405	550163	713668	527961	593520	528277	531487
Pentose	523804	417040	420550	387884	160460	190407	201582	200262	149486	150416	163898	166482	269739	269855	300938	225242	142518	156318	151765	149873	159457	129041	134553	147443
Pentose phosphate	176491	82557	110100	76496	92898	98208	122636	131241	98018	139253	133113	179009	60368	60594	67753	51948	34774	28160	27842	37132	140422	113063	132004	159519
PEP	82464	35468	54669	62311	29999	41233	46204	89023	47154	78272	68827	90755	16730	35487	50384	17904	14850	25602	31603	42487	72231	49423	51708	84056
Phenol Red	529964	481331	462027	545507	375305	472891	519406	545645	584670	550815	504187	589773	381040	468661	506426	385034	462101	528367	496847	595194	655802	633099	590500	843299
Phosphogluconate	83002	59899	60247	52009	22584	26421	22835	37265	22879	38630	32195	40502	35672	46313	55544	35267	9493	16558	21988	23916	37090	24642	23606	40376
Phosphogluconic Acid	85509	59899	60247	52968	22584	26421	22835	40912	22879	38630	30094	42807	40266	48838	56765	35267	9493	13236	22428	22784	37090	24642	27038	43441
Phosphoglyceric Acid	237235	119992	144246	165152	100900	163530	132871	298083	107985	190407	183114	217416	69082	139524	140932	75403	64574	78748	112582	158803	189851	138828	137848	243973
Proline	132103	112773	126946	104186	114102	121149	123076	116812	120867	124549	110608	123934	97007	78832	99654	67807	56817	41149	51891	41862	91864	85321	88286	103658
Quinic Acid	59462	44013	51659	86526	82748	48998	58302	45596	59103	179556	60454	52628	76722	74469	51349	99674	69774	77826	65005	72268	71859	84162	130009	47531
Seduheptulose-7-phosphate	427080	309921	350122	335498	98004	114544	146627	135417	150750	200177	163486	184467	244025	259922	312737	175512	76840	76760	81413	89369	213969	159678	172762	217993
Serine	659562	476112	558466	533995	444865	486965	531124	512024	306269	333343	312675	385924	401894	364687	366540	256648	285477	255836	264909	292394	294380	279992	247840	288540
Stearic Acid	615609	534184	525364	510662	570520	543606	560203	472450	498587	489123	566653	638767	627790	511651	501387	455010	433460	526776	540717	582336	458520	493533	490515	487530
Succinic Acid	416422	208378	310564	342333	111632	129745	117927	152512	219282	175909	213601	268921	333158	344058	297641	239831	104563	113232	97711	170799	182043	171980	195331	243360
Succinic-GSH	930773	754928	797913	780453	389944	671647	537138	811070	373674	566016	504351	545941	388818	675235	884358	484443	278145	282625	401219	431956	523879	410940	474459	566522
Taurine	15152334	12000021	14489756	12068878	4469507	3640031	5858496	4264058	6786172	6782866	5640582	6197108	13551039	11454646	12306518	9648989	4665512	4746916	4232683	3892172	5986053	5264281	6773268	5868020
Threonine	1602544	1380665	1483618	1468588	1436864	1285015	1589696	1245852	1334732	1505119	1266829	1473697	1222045	1007838	1138648	732251	652960	603967	617567	640876	1109156	1096809	1130459	1154041
Tyrosine	434714	441640	422528	435761	547592	527401	614358	537684	504622	567687	467279	526425	157431	156181	154452	103435	226547	191719	214060	223654	291974	284339	286490	361330
UDP	379397	212521	274043	254504	186523	240813	225634	287066	213004	210540	244595	259349	201865	259062	248196	216295	165552	173386	200317	201620	231151	221379	166425	223395
UDP-GlcNAc	19740574	13426879	16369004	14930371	3874731	4659947	6364619	6847348	5107493	7416184	6367045	7293974	8904025	11481825	15004340	6534458	2581519	2672325	3130267	3256257	7778300	5505639	5910920	7322553
UDP-glucose	2139876	1395633	1714553	1435666	474998	530674	632854	678729	2582143	3669065	3164476	3625370	1300718	1485346	2047951	1158124	372623	355646	391890	406178	4109012	2963298	3463207	4087035
UMP	111902	48815	73804	69134	45179	45463	55359	54303	54391	84099	71723	83081	58938	51893	80675	43909	19121	18861	13044	19090	94516	65108	58906	72306
Uracil	1827528	1346053	1478939	1355221	674321	712712	842613	838644	1464605	1452547	1499051	1580265	1342420	1403604	1466960	1051011	525438	529830	539444	586638	1774102	1476522	1425117	1626315
Uridine	308070	281713	268641	297133	2349693	2761514	2920161	2977613	177944	215422	178552	216786	222642	275776	248491	178118	1957841	2330106	2167868	2323961	193186	188321	163558	285673
UTP	121599	33843	83938	98841	21079	19488	29872	25903	74007	43414	51766	57870	154566	136183	68850	38225	28747	16531	24865	60757	107171	80696	54154	54138
